# Efficacy and safety of long-acting cabotegravir compared with daily oral tenofovir disoproxil fumarate plus emtricitabine to prevent HIV infection in cisgender men and transgender women who have sex with men 1 year after study unblinding: a secondary analysis of the phase 2b and 3 HPTN 083 randomised controlled trial

**DOI:** 10.1016/S2352-3018(23)00261-8

**Published:** 2023-11-09

**Authors:** Raphael J Landovitz, Brett S Hanscom, Meredith E Clement, Ha V Tran, Esper G Kallas, Manya Magnus, Omar Sued, Jorge Sanchez, Hyman Scott, Joe J Eron, Carlos del Rio, Sheldon D Fields, Mark A Marzinke, Susan H Eshleman, Deborah Donnell, Matthew A Spinelli, Ryan M Kofron, Richard Berman, Estelle M Piwowar-Manning, Paul A Richardson, Philip A Sullivan, Jonathan P Lucas, Peter L Anderson, Craig W Hendrix, Adeola Adeyeye, James F Rooney, Alex R Rinehart, Myron S Cohen, Marybeth McCauley, Beatriz Grinsztejn

**Affiliations:** Center for Clinical AIDS Research and Education, David Geffen School of Medicine, University of California Los Angeles, Los Angeles, CA, USA; Fred Hutchinson Cancer Research Center, Seattle, WA, USA; Louisiana State University Health Sciences Center, New Orleans, LA, USA; Department of Health Behavior, University of North Carolina at Chapel Hill, Chapel Hill, NC, USA; Department of Parasitic and Infectious Diseases, School of Medicine, University of Sao Paulo, Sao Paulo, Brazil; Department of Epidemiology, Milken Institute School of Public Health, George Washington University, Washington, DC, USA; Fundación Huésped, Buenos Aires, Argentina; Centro de Investigaciones Tecnologicas, Biomedicas y Medioambientales, Universidad Nacional Mayor de San Marcos, Lima, Peru; San Francisco Department of Public Health, San Francisco, CA, USA; Department of Health Behavior, University of North Carolina at Chapel Hill, Chapel Hill, NC, USA; Emory University School of Medicine and Grady Health System, Atlanta, GA, USA; Ross and Carol Nese College of Nursing, Pennsylvania State University, University Park, PA, USA; Johns Hopkins University School of Medicine, Baltimore, MD, USA; Johns Hopkins University School of Medicine, Baltimore, MD, USA; Fred Hutchinson Cancer Research Center, Seattle, WA, USA; Division of HIV, Infectious Diseases, and Global Medicine, University of California San Francisco, San Francisco, CA, USA; Center for Clinical AIDS Research and Education, David Geffen School of Medicine, University of California Los Angeles, Los Angeles, CA, USA; Fred Hutchinson Cancer Research Center, Seattle, WA, USA; Johns Hopkins University School of Medicine, Baltimore, MD, USA; Johns Hopkins University School of Medicine, Baltimore, MD, USA; Johns Hopkins University School of Medicine, Baltimore, MD, USA; FHI 360, Durham, NC, USA; Skaggs School of Pharmacy and Pharmaceutical Sciences, Anschutz Medical Campus, Aurora, CO, USA; Johns Hopkins University School of Medicine, Baltimore, MD, USA; Division of AIDS, National Institute of Allergy and Infectious Diseases, Bethesda, MD, USA; Gilead Sciences, Foster City, CA, USA; ViiV Healthcare, Research Triangle Park, NC, USA; Department of Health Behavior, University of North Carolina at Chapel Hill, Chapel Hill, NC, USA; FHI 360, Durham, NC, USA; Instituto Nacional de Infectologia Evandro Chagas-Fiocruz, Rio de Janeiro, Brazil

## Abstract

**Background:**

Injectable cabotegravir was superior to daily oral tenofovir disoproxil fumarate plus emtricitabine for HIV prevention in two clinical trials. Both trials had the primary aim of establishing the HIV prevention efficacy of long-acting injectable cabotegravir pre-exposure prophylaxis (PrEP) compared with tenofovir disoproxil fumarate plus emtricitabine daily oral PrEP. Long-acting PrEP was associated with diagnostic delays and integrase strand-transfer inhibitor (INSTI) resistance. This report presents findings from the first unblinded year of the HIV Prevention Trials Network (HPTN) 083 study.

**Methods:**

The HPTN 083 randomised controlled trial enrolled HIV-uninfected cisgender men and transgender women at elevated HIV risk who have sex with men, from 43 clinical research sites in Africa, Asia, Latin America, and the USA. Inclusion criteria included: a negative HIV serological test at the screening and study entry, undetectable HIV RNA levels within 14 days of study entry, age 18 years or older, overall good health as determined by clinical and laboratory evaluations, and a creatinine clearance of 60 mL/min or higher. Participants were randomly allocated to receive long-acting injectable cabotegravir or daily oral tenofovir disoproxil fumarate plus emtricitabine PrEP. After study unblinding, participants remained on their original regimen awaiting an extension study. HIV infections were characterised retrospectively at a central laboratory. Here we report the secondary analysis of efficacy and safety for the first unblinded year. The primary outcome was incident HIV infection. Efficacy analyses were done on the modified intention-to-treat population using a Cox regression model. Adverse events were compared across treatment groups and time periods (blinded *vs* unblinded). This trial is registered with ClinicalTrials.gov, NCT02720094.

**Findings:**

Of the 4488 participants who contributed person-time to the blinded analysis, 3290 contributed person-time to the first unblinded year analysis between May 15, 2020, and May 14, 2021. Updated HIV incidence in the blinded phase was 0·41 per 100 person-years for long-acting injectable cabotegravir PrEP and 1·29 per 100 person-years for daily oral tenofovir disoproxil fumarate plus emtricitabine PrEP (hazard ratio [HR] 0·31 [95% CI 0·17–0·58], p=0·0003). HIV incidence in the first unblinded year was 0·82 per 100 person-years for long-acting PrEP and 2·27 per 100 person-years for daily oral PrEP (HR 0·35 [0·18–0·69], p=0·002). Adherence to both study products decreased after study unblinding. Additional infections in the long-acting PrEP group included two with on-time injections; three with one or more delayed injections; two detected with long-acting PrEP reinitiation; and 11 more than 6 months after their last injection. Infection within 6 months of cabotegravir exposure was associated with diagnostic delays and INSTI resistance. Adverse events were generally consistent with previous reports; incident hypertension in the long-acting PrEP group requires further investigation.

**Interpretation:**

Long-acting injectable cabotegravir PrEP retained high efficacy for HIV prevention in men and transgender women who have sex with men during the first year of open-label follow-up, with a near-identical HR for HIV risk reduction between long-acting injectable cabotegravir and daily oral tenofovir disoproxil fumarate plus emtricitabine PrEP during the first year after unblinding compared with the blinded period. Extended follow-up further defined the risk period for diagnostic delays and emergence of INSTI resistance.

**Funding:**

Division of AIDS at the National Institute of Allergy and Infectious Diseases, ViiV Healthcare, and Gilead Sciences.

## Introduction

Long-acting injectable cabotegravir received US regulatory approval in 2021,^1^ and continues to receive non-US regulatory approval, for prevention of sexual HIV transmission across populations. Approvals were based on results from two randomised controlled trials (HIV Prevention Trials Network [HPTN] 083 and HPTN 084)^2,3^ evaluating HIV incidence in HIV-uninfected participants at elevated risk, who were randomly assigned to either long-acting injectable cabotegravir pre-exposure prophylaxis (PrEP) or daily oral tenofovir disoproxil fumarate plus emtricitabine PrEP. In these two studies, long-acting PrEP was found to be superior to daily oral PrEP for reducing HIV incidence, addressing an urgent need for more discreet HIV prevention strategies that are not dependent on adherence to daily pill taking.

HPTN 083 enrolled men and transgender women who have sex with men, and was unblinded in May, 2020 after efficacy was established at the first preplanned interim analysis. Previous reports include data from HPTN 083 before study unblinding.^2^ These publications reported a 66% reduced risk of HIV acquisition for long-acting compared with daily oral PrEP in the prespecified primary analysis.^2^ Long-acting PrEP was observed to delay detection of HIV infection by a median of 98 days (range 35–185) for incident infections,^4^ prompting a requirement in the US Food and Drug Administration labelling for inclusion of an RNA assay for HIV screening before each long-acting PrEP injection.^5^ Some infections in the long-acting PrEP group were found to have resistance-associated mutations (RAMs) that confer resistance to cabotegravir and other integrase strand-transfer inhibitors (INSTIs),^4,6^ potentially compromising the activity of first-line antiretroviral therapy (ART) containing dolutegravir or bictegravir.

After the study was unblinded, participants continued on their original randomly assigned study PrEP regimen until the protocol was amended and operationalised to offer eligible participants open-label long-acting PrEP in an ongoing open-label extension (OLE) study. Participants who were randomly assigned to long-acting PrEP were transitioned to daily oral PrEP during this period if they completed all of their prespecified long-acting PrEP doses or for adverse events while awaiting the OLE. This report presents additional efficacy and safety data for the first unblinded year of the study (up to May 15, 2021); participants began the OLE as local regulatory approvals for the amendment implementing the OLE were obtained, and the coverage of the OLE results in this report includes the first year after unblinding only. We also describe additional HIV infections that were identified in this extended analysis. This includes infections that occurred during the blinded phase of the trial but were not detected by the study sites until later in the study, and infections that occurred in the first year after study unblinding. We additionally present insights into the clinical contexts in which cabotegravir or INSTI RAMs have been identified.

## Methods

### Study design and participants

The HPTN 083 trial protocol was approved by the institutional review board, ethics committee, ministry of health, or a combination of these entities at each participating site, and the trial design has been previously described.^2^ Inclusion criteria were a negative rapid and laboratory-based HIV serological test at screening and study entry, undetectable HIV RNA within 14 days of study entry, age 18 years or older, overall good health as determined by clinical and laboratory evaluations, and a creatinine clearance of at least 60 mL/min. Participants were enrolled from 43 clinical research sites in Argentina, Brazil, Peru, South Africa, Thailand, the USA, and Viet Nam. Eligible participants were randomly assigned to long-acting injectable cabotegravir PrEP or daily oral tenofovir disoproxil fumarate plus emtricitabine PrEP, each with corresponding placebos. The study included a 5-week oral lead-in phase, followed by an oral-injectable comparison phase lasting approximately 3 years; participants who completed the 3-year comparison phase treatment or discontinued injections prematurely were offered 48 weeks of open-label daily oral PrEP. Cabotegravir was dosed as 30 mg/day orally or 600 mg (3 mL) intramuscularly, administered as a single gluteal injection at 8-week intervals after 4 weeks separating the first two injections. Oral PrEP was dosed as a single fixed dose formulation oral tablet of 300 mg of tenofovir disoproxil fumarate and 200 mg of emtricitabine daily. This report presents data from HIV infections that occurred during three periods: the blinded phase (infections that occurred before May 15, 2020); the first unblinded year (infections that occurred between May 16, 2020, and May 15, 2021, limited to infections that were detected by study sites before Nov 15, 2021); and the combined period (blinded phase plus first unblinded year). An updated CONSORT diagram showing participant dispositions in HPTN 083 that includes the first unblinded year is included in the appendix (p 2). The first site-positive visit refers to the first visit in which a reactive or positive HIV test result was obtained at the study site near the time of confirmed infection.^4^ The first HIV-positive visit refers to the first visit with confirmed HIV infection, based on real-time testing performed at study sites and retrospective testing performed at a central laboratory.^4^ All participants provided written informed consent. The HPTN 083 protocol was approved by the institutional review board, ethics committee, or ministry of health at each participating clinical research site.

### Randomisation and unblinded treatment continuation

At enrolment, participants were randomly assigned (1:1) to long-acting or daily oral PrEP as previously described.^2^ After study unblinding, participants continued on their originally randomly assigned active PrEP regimen without placebo dosing, but otherwise followed the original protocol-specified schedule of events (appendix p 3).

### Procedures

All study visits included one or two HIV rapid tests (of a variety of types cleared by the US Food and Drug Administration), a laboratory-based HIV antigen and antibody test, safety testing, storage of samples for retrospective analysis, and assessments for adverse events. Visits were conducted at 8-week intervals during the injection phase, and at four quarterly intervals for those discontinuing injections prematurely. Participants who arrived at the study site 8 weeks late or more for their injection were administered two injections 4 weeks apart, after which they returned to the 8-week injection schedule.

If an HIV assay performed at the study site was reactive or positive, stored samples were shipped to a central laboratory and analysed further to determine HIV infection status and the timing of infection.^4^ HIV genotyping was performed at a commercial laboratory for samples with viral loads greater than 500 copies per mL; INSTI resistance was assessed in samples with viral loads below 500 copies per mL using a low-viral-load single-genome sequencing assay.^6^

Plasma cabotegravir concentrations were measured in all samples from participants in the long-acting group with incident HIV infection. Plasma tenofovir and intraerythrocytic tenofovir diphosphate concentrations were measured in participants randomly assigned to the daily oral PrEP group who had incident infections, and in participants in the long-acting PrEP group who switched to daily oral PrEP. Tenofovir and tenofovir diphosphate concentrations were also determined in a previously described cohort of 390 randomly selected participants in the daily oral PrEP group to evaluate study product adherence.^2^ Drug concentration targets have been previously described.^4,7-9^

### Outcomes

The primary outcome was incident HIV infection. An independent adjudication endpoint committee confirmed HIV infections and determined the timing of infection using data from study sites and the central laboratory.^4^ Safety outcomes were grade 2 or higher adverse events. All adverse events were graded according to the Division of AIDS Table for Grading the Severity of Adult and Pediatric Adverse Events, corrected version 2.1.^10^

**Figure 1:**
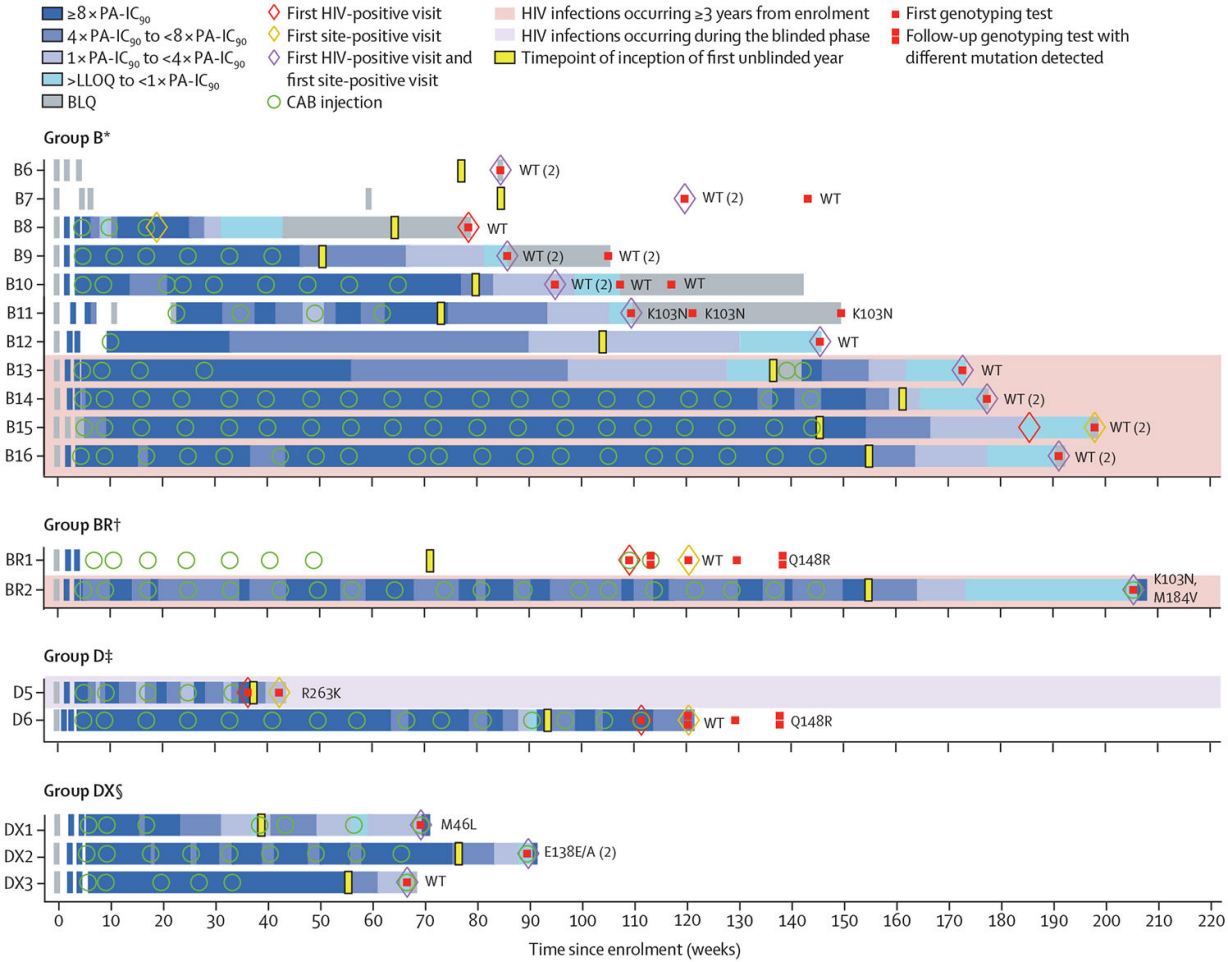
Pharmacological and virological data for HIV infections in the long-acting cabotegravir group identified in the extended analysis HIV genotyping results are shown to the right of each horizontal bar. Major resistance mutations are shown for nucleoside or nucleotide reverse-transcriptase inhibitors, non-nucleoside reverse-transcriptase inhibitors, protease inhibitors, and integrase strand-transfer inhibitors. The first visit with a genotyping result is shown with a single red square; later visits with genotyping results are shown with two red squares. The pink shading denotes participants for whom infections occurred more than 3 years from enrolment, and who were therefore excluded from prespecified efficacy analyses. BLQ=below the limit of quantification. CAB=cabotegravir. LLOQ=lower limit of quantification. PA-IC_90_=protein-adjusted 90% cabotegravir inhibitory concentration. WT=wild type. *Group B includes infections that occurred with no recent cabotegravir exposure (>6 months after the last long-acting cabotegravir injection). ^†^Group BR includes infections that occurred with no recent cabotegravir exposure (>6 months after the last cabotegravir injection) in participants who restarted long-acting cabotegravir before the study site detected the infection. ^‡^Group D includes infections that occurred despite on-time (<10 weeks between all) injections. §Group DX includes infections that occurred in participants who received at least one delayed cabotegravir injection (≥10 weeks between injections, but not meeting group B criteria).

Infections in the long-acting PrEP group were previously classified as follows:^2,4,11^ group A, infections present at study entry (prevalent or baseline); group B, infections detected more than 6 months after the last cabotegravir injection; group C, infections detected during the oral lead-in phase; group D, infections in the setting of on-time (no delays of longer than 10 weeks between 8-week injection intervals); group DX, infections in the setting of at least one inter-injection delay of 10 weeks or more but not meeting group B criteria; and group BR, infections detected more than 6 months after the last injection that were identified when long-acting PrEP was restarted in the open-label study. We used the term tail phase to describe infections that occurred more than 10 weeks but less than 66·6 weeks^12^ after a cabotegravir injection, and for which post-hoc pharmacological assessment suggested that the infection was likely to have been acquired while cabotegravir was quantifiable in plasma; this excludes participants re-exposed to injectable cabotegravir subsequent to infection. These include a subset of B and DX cases (figure 1).

### Statistical analysis

The efficacy analysis was done on the modified intention-to-treat population, excluding participants found to have HIV at enrolment, and used a Cox regression model, stratified by region, to estimate the hazard ratio (HR) of HIV infection in the long-acting versus daily oral PrEP group; 95% CIs and p values for the HR were based on the Wald statistic. Incidence rate 95% CIs were calculated by the Poisson distribution. SAS version 9.4 was used for analysis. Adverse event incidence rates were compared using negative-binomial regression allowing for multiple events per participant. A maximum of 3 years of follow-up time was included for each participant, corresponding to the maximum duration of active blinded treatment, with person-time censored at approximately 3 years after enrolment. Person-time inclusion for the first unblinded year analysis began at the final visit during the blinded phase and continued to the last visit with HIV test results before May 15, 2021, 3 years from enrolment, or entry into the OLE, whichever was earliest. For participants with incident HIV infection, the infection date was calculated as the midpoint between the first HIV-positive date and the last HIV-negative date. Infections occurring greater than 3 years after enrolment are described but were prespecified to be excluded from efficacy analyses.

This trial is registered with ClinicalTrials.gov, NCT02720094.

### Role of the funding source

The Division of AIDS of the National Institute of Allergy and Infectious Diseases provided regulatory sponsorship and primary funding for the trial, and was responsible for clinical monitoring of the trial. ViiV Healthcare provided additional funding and study product, and contributed to the design of the trial. Gilead Sciences provided study product for the trial.

## Results

As previously reported, 4566 participants were enrolled and constituted the intention-to-treat population of the blinded study. 3992 (87·4%) participants identified as men who have sex with men, with 570 (12·5%) identifying as transgender women who have sex with men (four participants preferred not to answer). Full ethnicity data are reported in the manuscript on the blinded phase of the study.^2^ Of the enrolled participants, 4488 contributed person-time to the blinded analysis. For the first year of unblinded follow-up, 3290 participants contributed person-time to the analysis. The combined analysis contains person-time contributions from 4492 participants (table 1). A summary of HIV infections identified through the first unblinded year is included in the appendix (p 4).

As described previously,^2,4^ PrEP agents, and injectable cabotegravir in particular, can delay detection of HIV infection using conventional HIV testing algorithms by inhibiting viral replication and delaying and diminishing antibody expression. In this analysis, we identified three additional incident HIV infections that occurred during the blinded phase of the study but were not detected until after study unblinding (one in the long-acting PrEP group and two in the daily oral PrEP group). Data from these three infections were combined with data from the original blinded analysis to obtain updated incidence rates in each group during the primary blinded analysis period. Based on this post-hoc analysis, 13 incident long-acting PrEP group infections were found in the blinded phase and 41 incident daily oral PrEP group infections in the blinded phase; these results are similar to those obtained in the previously reported primary analysis (table 1).^2^

We identified 44 additional incident HIV infections that occurred in the first unblinded year. This included 12 incident HIV infections in the long-acting PrEP group and 32 incident infections in the daily oral PrEP group (table 1). Five additional long-acting PrEP group infections are described but were excluded from efficacy analyses as they occurred more than 3 years after enrolment.

In the combined study period (blinded and first unblinded year), there were a total of 25 incident long-acting PrEP group infections and 73 incident daily oral PrEP group infections (table 1).

**Figure 2:**
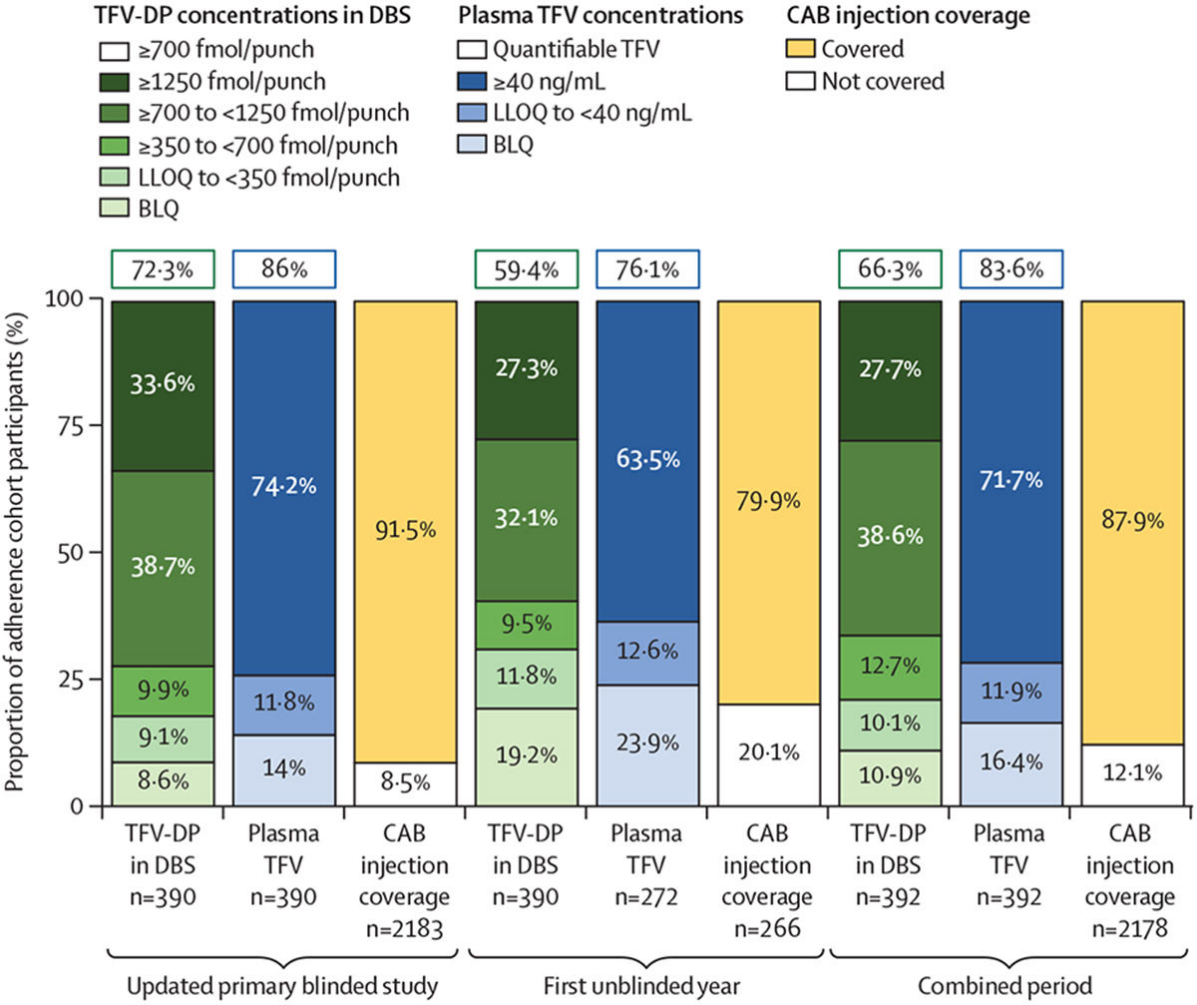
Study product adherence during the blinded phase, first unblinded year, and combined period Pharmacokinetic adherence testing was performed on samples collected at weeks 4, 9, 17, 33, 57, 81, 105, 129, 153, and 177, and on a quarterly schedule for 48 weeks and then annually if participants transitioned early to open-label tenofovir disoproxil fumarate plus emtricitabine. The median number of plasma and dried blood spots samples per participant was 5 (IQR 4–7) during the updated primary blinded study, 2 (1–2) during the first unblinded year, and 7 (5–8) during the combined period. The tenofovir diphosphate in dried blood bar shows the proportion of a randomly selected subset of the tenofovir disoproxil fumarate plus emtricitabine group participants with intraerythrocytic tenofovir diphosphate concentrations measured in dried blood spots in each adherence category; these data represent average dosing over the previous 1–2 months. The green boxed numbers above each histogram bar represent the aggregate of four or more doses per week; this dosing frequency is anticipated to provide high levels of rectal protection against HIV acquisition. Each participant selected for adherence testing could have up to eight samples included in this summary. Values for week 4 were adjusted for days on therapy, as steady-state drug concentrations were not yet achieved. The plasma tenofovir bar shows the proportion of a randomly selected subset of the tenofovir disoproxil fumarate plus emtricitabine group participants with plasma tenofovir in each adherence category; these data represent average dosing over the past 72 h. The blue boxed numbers above each histogram bar represent the aggregate of quantifiable tenofovir in plasma. Cabotegravir injection coverage was defined as an initial injection providing 6 weeks of anticipated protective coverage and subsequent injections providing 10 weeks of protective coverage. BLQ=below the limit of quantification. CAB=cabotegravir. DBS=dried blood spots. LLOQ=lower limit of quantification. TFV=tenofovir. TFV-DP=tenofovir diphosphate.

We previously reported that 91·5% of observed person-time was covered by cabotegravir injections during the blinded phase of the trial;^2^ coverage was defined by an initial injection providing 6 weeks of anticipated protective coverage and subsequent injections providing 10 weeks of anticipated coverage. During the first unblinded year and over the entire period of time since study inception, most person-time was covered by cabotegravir injections (figure 2).

We previously reported that during the blinded phase, nearly 75% of samples from the randomly selected adherence cohort had plasma tenofovir concentrations of at least 40 ng/mL, and most had quantifiable plasma tenofovir concentrations (lower limit of quantification 0·31 ng/mL); a little over 70% had tenofovir diphosphate concentrations of at least 700 fmol/punch (the equivalent of approximately four doses per week on average over the previous 1–2 months).^2^ After study unblinding, a little over half of evaluated samples had plasma tenofovir concentrations of at least 40 ng/mL, and over 75% of samples were quantifiable; nearly 60% of evaluated samples had tenofovir diphosphate concentrations of at least 700 fmol/punch. Across all evaluated samples (blinded and unblinded periods), plasma tenofovir concentrations were at least 40 ng/mL or quantifiable in a majority of the samples; tenofovir diphosphate concentrations were at least 700 fmol/punch in a little over 65% of evaluated samples (figure 2).

The decrease in adherence to daily oral PrEP in the first unblinded year is estimated to account for 35% of the observed increase in HIV incidence between the blinded study and the first unblinded year (appendix p 5). HIV incidence increased during the first unblinded year in all geographic regions (Africa, Asia, Latin America, and the USA), accompanied by a measurable decline in study product adherence; participants in Peru had the most pronounced drop in adherence and increase in HIV incidence (appendix p 6).

18 new injectable long-acting PrEP group infections are presented in this report (figure 1); additional details are provided in a previous report.^11^ These 18 cases include the 13 described above (one blinded, 12 unblinded) and five cases that occurred more than 3 years after enrolment (cases B13–16 and BR2; see below). The infection in the blinded phase occurred despite on-time injections (D5). The other 17 infections occurred in the first unblinded year. One infection occurred despite on-time injections and expected cabotegravir concentrations (D6), three occurred with at least one delay of more than 10 weeks between injections (DX1–3), and 11 occurred more than 6 months after the last cabotegravir administration (B6–16; this group includes two participants who never received cabotegravir injections). The remaining two infections were detected when long-acting PrEP was restarted after a 6-month or longer interruption (BR1 and BR2); in these cases, detection of infection was delayed at the study site, and the participants received long-acting PrEP on or after the first HIV-positive visit.

Participant D5 had on-time injections but was found to have consistent, rapid decay of cabotegravir concentrations between each injection; the participant’s BMI was 24 kg/m^2^, and no interacting concomitant medications or injection administration aberrancies were identified. The Arg263Lys INSTI mutation was identified at the first HIV-positive visit when the viral load was 59 copies per mL (figure 1). Case D6 had expected cabotegravir concentrations at most visits; four occurrences of trough cabotegravir concentrations between 4 and 8 times the protein-adjusted 90% cabotegravir inhibitory concentration (PA-IC_90_) and one trough concentration of approximately 1 times the PA-IC_90_ were observed later in the injection course (figure 1). Although wild-type virus was present at the first HIV-positive visit, Gln148Arg was identified 59 days later when the viral load was 9000 copies per mL. At the first HIV-positive visits, cabotegravir concentrations were less than 4 times the PA-IC_90_ (range below the limit of quantification to 495 ng/mL) in cases DX1–3. Cases DX1 and DX3 exhibited expected pharmacokinetics; case DX2 exhibited steep post-peak declines in cabotegravir concentrations and increased absorption after several cabotegravir injections.^11^ Case DX1 had multiple injection delays (figure 1). Cases DX1–3 did not have INSTI resistance mutations; DX3 probably represents infection acquired during the long-acting PrEP tail phase. Cases BR1 and BR2, while technically tail-phase infections, represent HIV acquisition detected by the site after long-acting PrEP re-exposure, and therefore have different implications for INSTI resistance and HIV diagnostic assay delays. Case BR1 did not have resistance mutations at the first HIV-positive visit but developed the Gln148Arg mutation before the site detected the infection; the detection of this mutation was transient. Case BR2 did not have INSTI resistance mutations, but the Lys103Asn and Met184Val mutations were detected at the first HIV-positive visit, probably due to transmitted resistance. Cases B9–11 and B13–16 did not have INSTI resistance mutations; in these cases, HIV infection was probably acquired during the tail phase (figure 1). Of note, participant B12 started antiretroviral treatment before the first HIV-positive visit; this was also the site-positive visit. Additional pharmacological and virological details of these cases have been reported previously.^11^

**Figure 3:**
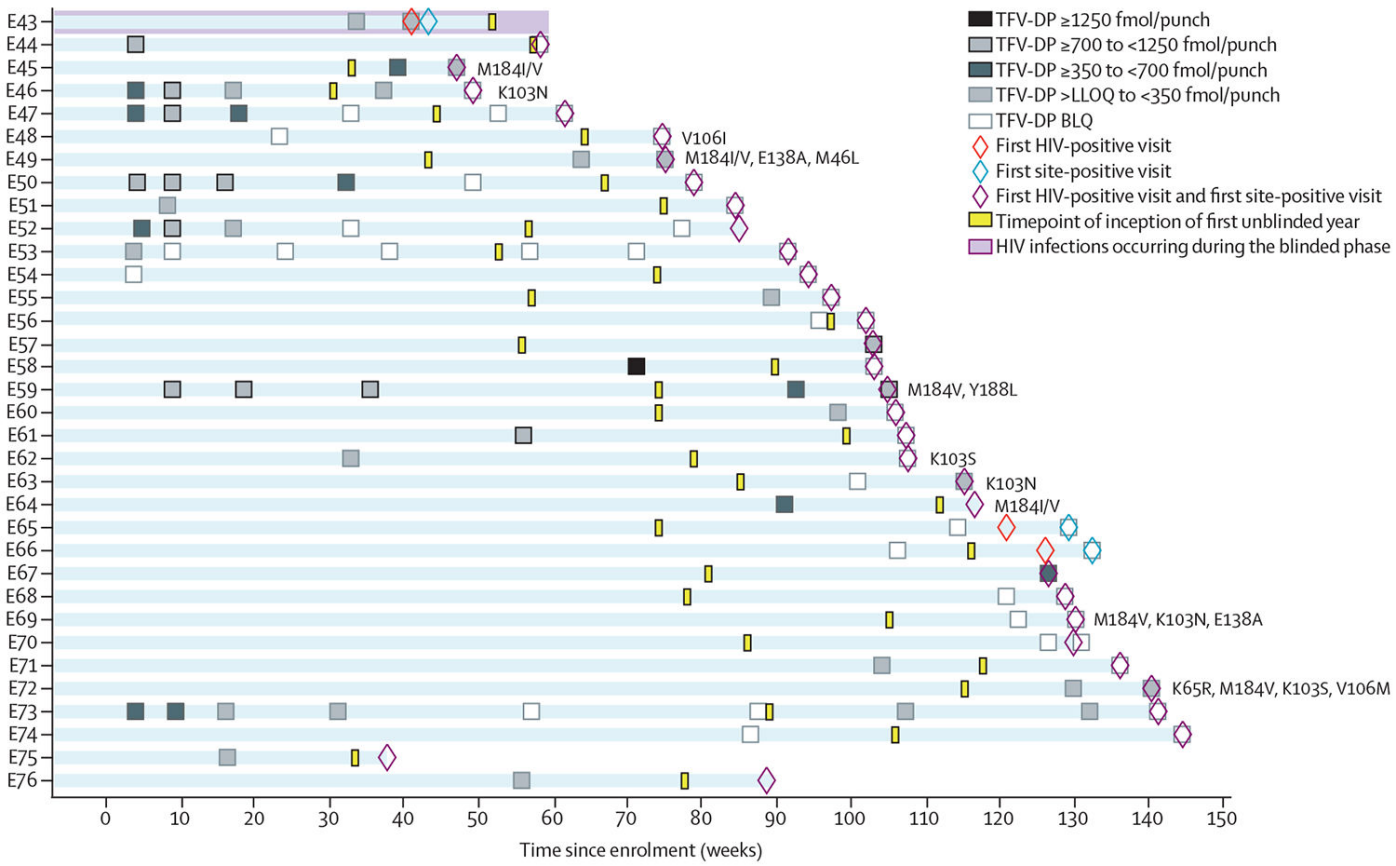
Pharmacological and virological data for HIV infections in the oral tenofovir disoproxil fumarate plus emtricitabine group that were identified in the extended analysis HIV genotyping results are shown for the first visit with a viral load greater than 500 copies per mL. HIV genotyping results are shown to the right of each bar. These include major mutations that confer resistance to nucleoside or nucleotide reverse transcriptase inhibitors, non-nucleoside reverse transcriptase inhibitors, and protease inhibitors. BLQ=below the limit of quantification. LLOQ=lower limit of quantification. TFV-DP=tenofovir diphosphate.

34 new daily oral PrEP group infections are presented in this report (two in the blinded phase and 32 in the first unblinded year; figure 3). None of the 34 participants had pharmacological data consistent with daily oral PrEP use. Two cases appear to have occurred in participants who were taking an average of four or more doses per week (E57 and E59). Case E59 had reverse transcriptase mutations Met184Val and Tyr188Leu at the first HIV-positive visit; case E57 was not genotyped due to low viral load. Two additional cases (E64 and E67) had evidence of recent partial adherence (two to three doses per week on average); E64 also had Met184Ile/Val. E67 was not genotyped due to low viral load. Additional pharmacological and virological details of these cases have been reported previously.^11^

Types, incidence rates, and differences between study groups of grade 2 and higher adverse events were generally consistent with the results from the blinded phase of the study; statistically significant increased rates of hypertension, total and LDL cholesterol, malaise, and proctitis were newly observed in the long-acting PrEP group (table 2). Injection site reactions were reported at roughly half the rate observed in the blinded phase, and did not lead to any long-acting PrEP discontinuations in the first unblinded year (appendix p 7). Previously reported similarities in annual rates of weight gain observed subsequent to the first year of the randomised comparison (1·11 kg/year [95% CI 0·82–1·41] in the long-acting PrEP group, and 1·19 kg/year [0·90–1·49] in the daily oral PrEP group) were sustained at rates of 0·84 kg/year (0·54–1·14) in the long-acting PrEP group and 0·80 kg/year (0·5–1·11) in the daily oral PrEP group (p=0·87). Differences in weight change accrued in the first year of exposure, largely driven by weight loss of −0·50 kg/year in the daily oral PrEP group, resulted in an overall (combined blinded and unblinded periods) excess in median weight gain of 0·94 kg (0·05–1·82) for the long-acting PrEP group participants (appendix p 8).

## Discussion

Long-acting injectable cabotegravir PrEP was shown to be superior to daily oral tenofovir disoproxil fumarate plus emtricitabine PrEP for reducing HIV incidence in two large randomised controlled clinical trials, resulting in US and non-US regulatory approvals beginning in December, 2020, with additional regulatory submissions pending. An additional year comparing long-acting with daily oral PrEP after study unblinding added approximately 50% more observation time and extended the median follow-up time by over a year. These additional data reaffirmed the relative efficacy observed in the primary study report, showing a consistent reduction in risk of infection by roughly two-thirds for long-acting compared with daily oral PrEP in men and transgender women who have sex with men. Differences in total and LDL cholesterol are expected based on the lipid-suppressive effects of tenofovir disoproxil fumarate. The observed increased rates of new hypertension in the long-acting PrEP group require additional interrogation; there is an increasing body of literature suggesting an association of INSTIs with hypertension, dysglycaemia, and cardiovascular risk, which warrants vigilance as cabotegravir is scaled up as a prevention agent.^13^ Injection site reactions continued to decrease over time and discontinuations attributable to injection-related events remained rare. Weight gain continued at equal rates between groups, although a persistent excess in overall weight increases remained in long-acting PrEP group participants, probably attributable to the early anorectic effect of oral tenofovir disoproxil fumarate plus emtricitabine start-up. The long-term implications of weight gain and effects on overall cardiovascular risk require further investigation. Research on long-acting PrEP is now pivoting from efficacy to longer-term safety, acceptability, correlates of protection, implications of resistance, and implementation through a lens of global equity.

The extended analysis in this report supports our previous studies, showing the potential of long-acting PrEP to delay detection of HIV infections using conventional HIV testing algorithms based solely on antibody and antigen-based testing. These delays are due to potent suppression of viral replication with delayed and diminished antigen detection and antibody production. Importantly, the correlates of HIV protection with long-acting PrEP remain to be defined; rare breakthrough infections have been observed at high plasma cabotegravir concentrations (>8 times the PA-IC_90_). This research requires precise determination of the timing of HIV acquisition and determination of plasma, intracellular, and tissue drug concentrations at the time of exposure.

Tenofovir disoproxil fumarate plus emtricitabine breakthrough infections are rare in the setting of high adherence to daily oral PrEP; almost all of the infections observed in the daily oral PrEP group of HPTN 083 occurred in participants with poor adherence or no evidence of drug dosing. Because the active forms of tenofovir and emtricitabine have relatively short plasma half-lives, infections in people taking daily oral PrEP can usually be detected with little or no delay using routine testing algorithms, apart from in the window or eclipse phase of early acute HIV infection. In fact, all detection delays in participants in the daily oral PrEP group of HPTN 083 were due to detection of the infection during the acute phase (ie, Fiebig Stage I^14^), which reflected frequent study visits.

We identified three additional incident HIV infections during retrospective testing that occurred during the original blinded study period. Inclusion of these three cases with the primary data from the blinded phase of the study did not alter the direction, magnitude, or overall interpretation of the primary study results. We have now identified six cabotegravir infections to date that occurred despite on-time injections, including the two cases described in this report; major INSTI RAMs were identified in all six cases. Although these six cases are of great interest, it is important to note that they occurred among 2282 participants randomly assigned to long-acting PrEP. Importantly, all six of these participants had virological suppression using non-nucleoside reverse transcriptase inhibitors or boosted protease inhibitor-based ART. A key unanswered clinical management question is whether these long-acting PrEP breakthrough infections can be effectively treated with dolutegravir or bictegravir-containing ART. Further data from ongoing cohorts and from clinical experience will provide useful insights into this issue.

Although the majority of breakthrough infections with on-time cabotegravir injections to date have had expected plasma cabotegravir concentrations, case D5 uniquely exhibited consistently low steady-state trough cabotegravir concentrations; the pattern of cabotegravir concentrations in this case was distinct from the pattern in other D group participants, who had lower cabotegravir concentrations between the first and second injections, or wide cabotegravir trough variability over multiple injections. The observed concentrations for this case might be attributed to an increased absorption rate constant (k_a_), which was observed after each injection.^11^ Of note, injectable cabotegravir exhibits so-called flip-flop kinetics, in which absorption of the drug from the intramuscular depot is the primary driver of the terminal phase decay of the drug concentration–time profile.^15,16^ Individuals with lower BMIs tend to have accelerated (high) absorption rate constants; however, this participant’s BMI (24·1 kg/m^2^) does not explain the more rapid absorption of injectable cabotegravir from the intramuscular depot. Furthermore, based on a population pharmacokinetic model, sex at birth and BMI contribute only 9% to inter-individual pharmacokinetic variability of injectable cabotegravir.^12,17^ Aberrations during product administration were not noted, and concomitant medications that could impact cabotegravir pharmacology were not identified. Similar post-injection declines were observed in two other cases (DX2 and B11), and cabotegravir drug concentrations were unquantifiable at the first HIV-positive visits for these participants. It is unclear how frequently this rapid decay pattern occurred in study participants who did not acquire HIV on study, or whether it played a role in cabotegravir breakthrough.

This report includes three long-acting PrEP group infections that occurred in the setting of injection delays (the DX cases, not observed during analysis of the blinded phase). These cases, along with the full set of B cases (incident infections that occurred more than 6 months after the last cabotegravir exposure), demonstrate that HIV infections that occurred more than 6 months after the last cabotegravir injection did not result in INSTI resistance and were usually detected without delay using standard testing algorithms. Although exact timing of tail-phase infection acquisition cannot be determined, data from HPTN 077 suggest that cabotegravir concentrations were likely to be quantifiable in the time period leading to the first HIV-positive visit;^12^ this includes cases DX3, B9–11, and B13–16. Further interrogation of tail-phase kinetics could be helpful in understanding the correlates of INSTI resistance with cabotegravir concentration decay.

Importantly, long-acting PrEP correlates or thresholds of protection might be distinct for rectal, vaginal, penile, and parenteral exposures, and are yet to be defined. Further analysis of HIV infections that occurred after the first unblinded year and inclusion of cases from HPTN 084 in cisgender women will hopefully provide additional insights into these correlates of protection with different routes of exposure. Defining the pharmacological correlates of protection is essential to determining the optimal dosing interval, the impact of delayed injections, and the time to onset of protective efficacy. This information will also inform clinical use of long-acting PrEP.

The first unblinded year of HPTN 083 was characterised by increased HIV incidence rates in both groups compared with the blinded period, with a consistent risk reduction for long-acting compared with daily oral PrEP; this was partly due to decreased study product adherence in both groups. These findings were observed across sites and regions, although the greatest decrease in product adherence was observed in Peru. The increased incidence rates and decreased adherence metrics in both groups are a sobering reminder that although long-acting PrEP might remove some barriers to successful HIV prevention by eliminating the need for daily oral dosing, it will not, on its own, remedy all challenges associated with PrEP uptake, adherence, and persistence that are required to end the global HIV epidemic. The HIV epidemic in Peru, concentrated disproportionately in cisgender men and transgender women who have sex with men, requires urgent action with a variety of biomedical HIV prevention options, structural interventions, and support services aimed at addressing inequities and social determinants of health. In ImPrEP, a large implementation study of oral tenofovir disoproxil fumarate plus emtricitabine PrEP conducted in Brazil, Mexico, and Peru, participants from Peru had greater HIV incidence, as well as increased odds of early loss to follow-up, lower odds of PrEP adherence, and longer-term PrEP engagement, which recent literature suggests could be related to a lower level of PrEP awareness and willingness.^18^ In a recent study, the annualised HIV incidence in Peru, using the Maxim HIV-1 Lag-Avidity EIA assay as part of a recent infection testing algorithm, was 6·69 (95% CI 5·57–7·81), and higher among those aged 18–24 years at 9·77 (7·34–12·20).^19^ These data highlight the urgent need for additional research to interrogate observed increases in HIV incidence, odds of early loss to follow-up, PrEP adherence, and long-term engagement.

This study has several limitations, including the inability to fully explain the increase in proportional incidence in both groups, incomplete biomarkers of adherence to daily oral PrEP complicating the explanation of its failures, and the small cumulative number of long-acting PrEP failures and delays in HIV detection making evaluation of the pharmacological correlates of protection elusive.

HPTN 083 continues in an OLE phase in which eligible participants are offered open-label long-acting or daily oral PrEP. Participants initiating cabotegravir for the first time have the option to exclude the oral lead-in period. The OLE will provide helpful data for assessing the current US Food and Drug Administration prescribing information and US Centers for Disease Control and Prevention guidance, in which the oral lead-in phase is considered to be optional.^5,20^ Additionally, the HPTN 083 OLE includes the use of an HIV RNA assay for HIV screening before starting long-acting PrEP and at every injection visit. Data from the extension study will allow systematic evaluation of risks and potential benefits of HIV RNA screening (eg, earlier detection of infections with the potential to avoid INSTI resistance contrasted with substantially increased cost, operational complexity, and risk of false-positive test results).

Long-acting PrEP is a novel, highly effective HIV prevention strategy across at-risk populations.^2,3^ An additional year of open-label follow-up in HPTN 083 demonstrated consistent and sustained HIV prevention benefits for long-acting over daily oral PrEP and provided additional information on cabotegravir pharmacokinetics and infections that occurred in participants who received long-acting PrEP; this includes noting the absence of diagnostic delays and INSTI resistance when incident infection occurred more than 6 months after the last injection, including patients who had reexposure to injectable cabotegravir. Of 2246 participants enrolled in the long-acting PrEP group, there were 30 incident HIV infections detected up to the end of the first unblinded year in a variety of clinical scenarios; of these, nine (30%) had INSTI-associated RAMs. Delayed initiation of ART when HIV infection is detected with the use of long-acting PrEP could lead to the accumulation of new or additional INSTI RAMs. Important issues for implementation will be further informed by the ongoing HPTN 083 open-label study and the parallel open-label extension study in HPTN 084 in cisgender women.

## Supplementary Material

1

## Figures and Tables

**Table 1:** HIV incidence overall, by treatment group, and by analysis period

	Long-acting cabotegravir group	Daily oral tenofovir disoproxil fumarate plus emtricitabine group	HR (95% CI)	p value
Blinded phase (updated analysis)	..	..	0·31 (0·17–0·58)	0·0003
Number of participants	2241	2247	..	..
Median (IQR) time on study, days	511 (314–700)	511 (307–700)	..	..
Infections/person-years	13/3204	41/3186	..	..
Incidence rate per 100 person-years (95% CI)	0·41 (0·22–0·69)	1·29 (0·92–1·75)	..	..
First unblinded year analysis	..	..	0·35 (0·18–0·69)	0·0021
Number of participants	1663	1627	..	..
Median (IQR) time on study, days	344 (275–380)	342 (279–378)	..	..
Infections/person-years	12/1456	32/1410	..	..
Incidence rate per 100 person-years (95% CI)	0·82 (0·43–1·44)	2·27 (1·55–3·20)	..	..
Combined period	..	..	0·34 (0·22–0·53)	<0·0001
Number of participants	2244	2248	..	..
Median (IQR) time on study, days	823 (596–1026)	808 (577–1015)	..	..
Infections/person-years	25/4660	73/4596	..	..
Incidence rate per 100 person-years (95% CI)	0·54 (0·35–0·79)	1·59 (1·25–2·00)	..	..

HR=hazard ratio.

**Table 2:** Incidence rates of adverse events during the blinded phase and first unblinded year

	Overall		Daily oral tenofovir disoproxil fumarate plus emtricitabine group		Long-acting cabotegravir group		p value
	Events	Incidence rate, per 100 person-years	Events	Incidence rate, per 100 person-years	Events	Incidence rate, per 100 person-years	
All grade ≥2 adverse events*	7479	260·2 (254·4–266·2)	3678	257·6 (249·3–266·0)	3801	262·9 (254·6–271·4)	0·38
Most common grade ≥2 adverse events							
Decreased creatinine clearance	1869	65·03 (62·12–68·05)	977	68·43 (64·21–72·86)	892	61·67 (57·69–65·86)	0·02
Diarrhoea†‡	51	1·77 (1·32–2·33)	23	1·61 (1·02–2·42)	28	1·94 (1·29–2·80)	0·53
Headache^‡^	119	4·14 (3·43–4·95)	51	3·57 (2·66–4·70)	68	4·70 (3·65–5·96)	0·21
Hypoglycaemia^‡^	57	1·98 (1·50–2·57)	34	2·38 (1·65–3·33)	23	1·59 (1·01–2·39)	0·15
Increased alanine aminotransferase	139	4·84 (4·07–5·71)	67	4·69 (3·64–5·96)	72	4·98 (3·90–6·27)	0·75
Increased amylase	108	3·76 (3·08–4·54)	60	4·20 (3·21–5·41)	48	3·32 (2·45–4·40)	0·26
Increased aspartate aminotransferase	112	3·90 (3·21–4·69)	55	3·85 (2·90–5·01)	57	3·94 (2·98–5·11)	0·90
Increased blood glucose	100	3·48 (2·83–4·23)	40	2·80 (2·00–3·82)	60	4·15 (3·17–5·34)	0·06
Increased creatine kinase	280	9·74 (8·63–10·95)	141	9·88 (8·31–11·65)	139	9·61 (8·08–11·35)	0·83
Increased lipase	153	5·32 (4·51–6·24)	82	5·74 (4·57–7·13)	71	4·91 (3·83–6·19)	0·41
Increased serum creatinine	320	11·13 (9·95–12·42)	166	11·63 (9·93–13·54)	154	10·65 (9·03–12·47)	0·54
Musculoskeletal discomfort‡	143	4·98 (4·19–5·86)	75	5·25 (4·13–6·59)	68	4·70 (3·65–5·96)	0·52
Nasopharyngitis^‡^	165	5·74 (4·90–6·69)	72	5·04 (3·95–6·35)	93	6·43 (5·19–7·88)	0·16
Pyrexia	53	1·84 (1·38–2·41)	16	1·12 (0·64–1·82)	37	2·56 (1·80–3·53)	<0·0001
Rash‡	85	2·96 (2·36–3·66)	43	3·01 (2·18–4·06)	42	2·90 (2·09–3·93)	0·90
Upper respiratory infection‡	39	1·36 (0·96–1·86)	17	1·19 (0·69–1·91)	22	1·52 (0·95–2·30)	0·45
Additional statistically significant grade ≥2 adverse events reported during the first unblinded year‡							
Increased blood pressure	21	0·73 (0·45–1·12)	2	0·14 (0·02–0·51)	19	1·31 (0·79–2·05)	<0·0001
Malaise	18	0·63 (0·37–0·99)	2	0·14 (0·02–0·51)	16	1·11 (0·63–1·80)	<0·0001
Increased total cholesterol	47	1·64 (1·20–2·17)	14	0·98 (0·54–1·65)	33	2·28 (1·57–3·20)	<0·0001
Increased LDL cholesterol	64	2·23 (1·71–2·84)	23	1·61 (1·02–2·42)	41	2·83 (2·03–3·85)	0·03
Proctitis	22	0·77 (0·48–1·16)	6	0·42 (0·15–0·91)	16	1·11 (0·63–1·80)	0·04
Adverse events of special interest							
Seizure	1	0·03 (0·00–0·19)	1	0·07 (0·00–0·39)	0	0·00 (0·00–0·00)	0·97
Liver-related adverse events resulting in discontinuation of study products	34	1·31 (0·90–1·82)	15	1·14 (0·64–1·88)	19	1·47 (0·89–2·30)	0·38
Injection site reactions grade ≥2							
Blinded phase	..	..	..	..	1009 (47·7%)	..	..
First unblinded year	..	..	..	..	332 (22·9%)	..	..

Data are n, n (%), incident rate (95% CI), or p value. MedDRA=Medical Dictionary for Regulatory Activities, version 23.1. *Included are only adverse events that were assigned MedDRA terms by clinical staff. Injection site reactions and sexually transmitted infections are not included with adverse events. Inappropriately enrolled participants and participants who did not receive any oral trial drug are excluded. †This adverse event category combines multiple MedDRA terms that were too similar to report individually. ‡Only adverse events with a statistically significant difference between long-acting and daily oral pre-exposure prophylaxis groups (p<0·05) are shown.

## Data Availability

We will make de-identified individual-level data presented in this manuscript available upon approval of a request, by network and National Institute of Allergy and Infectious Diseases policy, 1 year after database lock. Research related to primary and secondary endpoints will have priority for investigation by study team members. A data archive will be held at the Fred Hutch Cancer Center, Seattle, WA, USA. Requests can be sent to HPTN-Data-Access@scharp.org.

## Research in context

### Evidence before this study

We searched ClinicalTrials.gov and PubMed from inception to May 3, 2023, using the search terms “cabotegravir” AND “prevention” or “efficacy”, “safety”, or “resistance”. Our search yielded 96 publications and five ongoing trials. Long-acting injectable cabotegravir is a novel integrase strand-transfer inhibitor (INSTI) that has been combined with long-acting injectable rilpivirine to successfully maintain HIV virological suppression in people with HIV. Observational data from 2023 also provocatively suggest the possibility of using this combination even in people with HIV with viraemia. Cabotegravir as a single agent has been studied for HIV prevention in comparison with daily oral tenofovir disoproxil fumarate plus emtricitabine in two randomised, active controlled, double-blind, double-dummy, registrational clinical trials (HIV Prevention Trials Network [HPTN] studies 083 and 084). The blinded parts of both studies were stopped early and demonstrated that long-acting injectable cabotegravir pre-exposure prophylaxis (PrEP) was superior to daily oral tenofovir disoproxil fumarate plus emtricitabine PrEP for HIV prevention in cisgender men and transgender women who have sex with men (HPTN 083) and in cisgender women in southern and eastern Africa (HPTN 084). HPTN 083 and 084 demonstrated 66% and 89% reductions in incident HIV infections for long-acting compared with daily oral PrEP in their respective populations. Both studies demonstrated that long-acting antiviral agents, in the context of either PrEP initiation with undiagnosed HIV infection or in PrEP breakthrough, delay such infection detection using conventional HIV diagnostic assays as a consequence of suppressed viraemia and delayed and attenuated antigen and antibody production. In the setting of on-time injection administration, rare failures were associated with INSTI resistance with the potential to compromise global first-line HIV treatment regimens. A priori concerns that HIV acquisition during the prolonged pharmacokinetic tail phase after a terminal injection would lead to INSTI resistance were not supported by the limited clinical trial experience.

### Added value of this study

This study is the first analysis of HPTN 083 subsequent to study unblinding for efficacy in May, 2020, providing an additional 2866 person-years of follow-up from participants remaining on their original randomised study treatment. We describe the persistence of the 66% reduction in incident HIV in individuals originally randomly assigned to long-acting compared with daily oral PrEP, despite increases in HIV incidence observed in both treatment groups. We characterise 52 additional HIV infections (18 in the long-acting PrEP group and 34 in the daily oral PrEP group) with regard to PrEP drug exposures, resistance, and clinical context; detailed virology and pharmacology from these cases are in a companion publication. We are now able to make observations on the clinical contexts in which long-acting PrEP group infections occur, their HIV diagnostic testing characteristics and behaviour, and the probability of selecting for INSTI resistance mutations. We further describe the adverse event profile of cabotegravir, including an increased rate of incident hypertension that requires further investigation.

### Implications of all the available evidence

Injectable cabotegravir has now obtained regulatory approval in the USA and an increasing number of other countries for the prevention of HIV sexual transmission. The totality of evidence to date supports high levels of protective efficacy with good tolerability across populations and geographies considered at sexual risk. The consistency of the relative advantage of long-acting compared with daily oral PrEP is likely to be attributable to better coverage of sex acts without requiring consumer-initiated daily ingestion. Although PrEP failures are extremely rare, breakthroughs despite on-time injections remain unexplained and are associated with INSTI resistance. For individuals born male, HIV acquisition 6 or more months after terminal cabotegravir injection does not appear to be associated with either INSTI resistance or delays in breakthrough detection using conventional HIV diagnostic assays. The field is now pivoting to evaluating implementation strategies for the global scale-up of long-acting injectable cabotegravir PrEP as part of HIV prevention programmes, in addition to interrogating existing and ongoing data to identify the drug-concentration correlates of protection, the risks and benefits of using an oral lead-in before long-acting injection, and the performance characteristics of sensitive HIV RNA testing as part of HIV screening algorithms.
