# Supplementary material for: Efficacy and safety of long-acting cabotegravir compared with daily oral tenofovir disoproxil fumarate plus emtricitabine to prevent HIV infection in cisgender men and transgender women who have sex with men 1 year after study unblinding: a secondary analysis of the phase 2b and 3 HPTN 083 randomised controlled trial
Source: Lancet HIV. Author manuscript; Available in PMC 2024 Sep 5. (PMC11375758; doi:10.1016/S2352-3018(23)00261-8)
Supplement: 1 [file NIHMS2017279-supplement-1.pdf]

THE LANCET
HIV

Supplementary appendix

This appendix formed part of the original submission and has been peer reviewed. We post it as supplied by the authors.

Supplement to: Landovitz RJ, Hanscom BS, Clement ME, et al. Efficacy and safety of long-acting cabotegravir compared with daily oral tenofovir disoproxil fumarate plus emtricitabine to prevent HIV infection in cisgender men and transgender women who have sex with men 1 year after study unblinding: a secondary analysis of the phase 2b and 3 HPTN 083 randomised controlled trial. *Lancet HIV* 2023; published online Nov 9. https://doi.org/10.1016/S2352-3018(23)00261-8.

Table of Contents

Supplementary Figure 1: HPTN 083 Consort Diagram 2

Supplementary Figure 2: Timeline of HPTN 083 from Enrollment to the End of the First Unblinded Year 3

Supplementary Table 1: Summary of HIV Infections 4

Supplementary Table 2: Estimated Increase in HIV Incidence Due to Decreased TDF/FTC Adherence 5

Supplementary Table 3: Changing Adherence Patterns: Plasma TFV and TFV-DP 6

Supplementary Table 4: Adverse Events Leading to Study Product Discontinuation during the First Unblinded Year by Study Arm 7

Supplementary Figure 3: Median Changes in Weight (kg) from Enrollment (W0) to Week 153 (W153) 8

Supplementary Acknowledgements 9

Supplementary Figure 1: HPTN 083 Consort Diagram

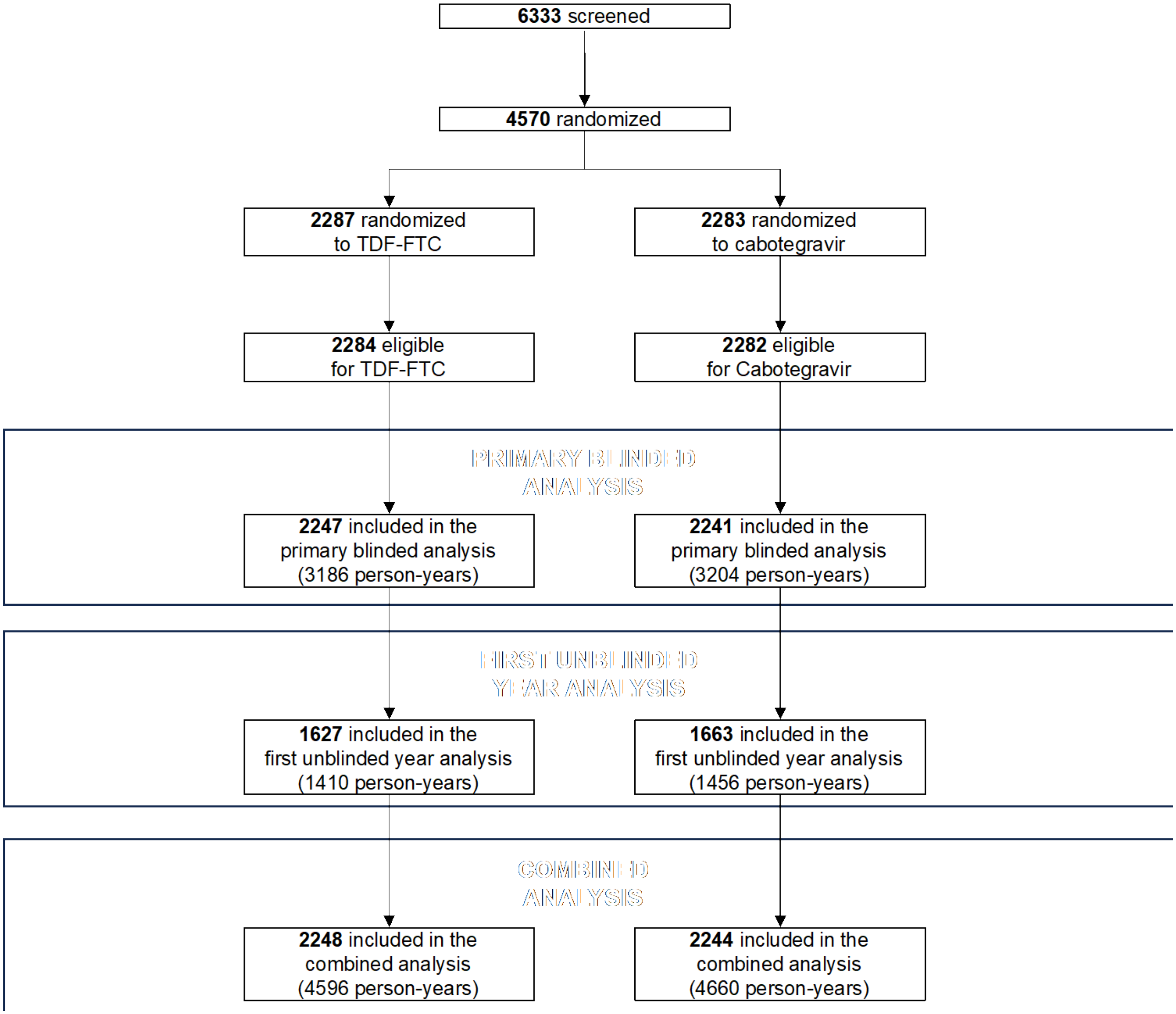

Supplementary Figure 2: Timeline of HPTN 083 from Enrollment to the End of the First Unblinded Year

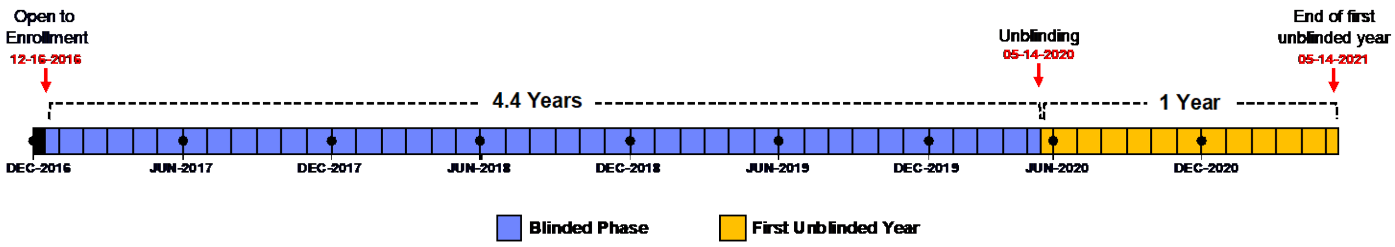

Supplementary Table 1: Summary of HIV Infections

Fifty-two additional HIV infections are described in this report (18 CAB arm, 34 TDF/FTC arm). Three of these cases occurred in the blinded phase of the study (1 CAB 2 TDF/FTC arm) and 49 occurred in the first unblinded year (17 CAB arm, 32 TDF/FTC arm). The table below summarizes the timing of HIV infections and the number of cases included in the assessment of HIV incidence.

	Total # cases	# incident cases	# cases included in the HIV incidence assessment[†]
Blinded phase (updated)			
CAB arm	17	13[¶]	13[¶]
TDF/FTC arm	44	41	41
First unblinded year			
CAB arm	17	17	12[‡]
TDF/FTC arm	32	32	32
Total (updated analysis)			
CAB arm	34	30	25[‡]
TDF/FTC arm	73	73	73

[†] This group is limited to incident infections that occurred <3 years after study initiation.
[‡] Excludes five infections that occurred >3 years after study initiation.
[¶] One prevalent (baseline) infection in the CAB arm was initially characterized as an incident infection and was included in the HIV incidence assessment in the primary study report; that case is not included here.

Supplementary Table 2: Estimated Increase in HIV Incidence Due to Decreased TDF/FTC Adherence

Blinded phase	
Quantifiable TFV in plasma	0.86
Predicted RR (TDF/FTC vs Placebo), meta-regression	0.256
Observed TDF/FTC arm incidence	1.29
Predicted placebo incidence (observed incidence / predicted RR)	5.04
First unblinded year	
Quantifiable TFV in plasma	0.76
Predicted RR (TDF/FTC vs Placebo), meta-regression	0.327
Observed TDF/FTC arm incidence	2.3
Predicted placebo incidence (observed incidence / predicted RR)	7.03
Predicted first unblinded year incidence based on estimated blinded phase background incidence	1.65
Estimated proportion of HIV incidence attributed to decreased adherence	35%*

Abbreviations: HIV, human immunodeficiency virus; TDF, tenofovir disoproxil fumarate; FTC, emtricitabine; TFV, tenofovir; RR, relative risk

* Assuming background incidence remained at 5.04 during the first unblinded year, observed TDF/FTC adherence would predict a 1-0.327 = 67% risk reduction in the TDF/FTC arm, yielding an incidence rate of 1.65. The observed TDF/FTC incidence in year was 2.3, suggesting that decreased adherence explained just (1.65-1.29)/(2.3-1.29)x100 = 35% of the increase.

Supplementary Table 3: Changing Adherence Patterns: Plasma TFV and TFV-DP

	Mean TFV (ng/mL)			Mean TFV-DP (fmol/punch)		
	Blinded phase	First unblinded year	% Change	Blinded phase	First unblinded year	% Change
Argentina	85	92	8%	1335	1289	-3%
Brazil	118	101	-14%	1064	987	-7%
Peru	58	35	-4%	485	269	-45%
South Africa	77	52	-32%	844	809	-4%
Thailand	72	70	-2%	1036	896	-14%
Vietnam	81	77	-5%	978	700	-28%
US Black	110	128	16%	822	748	-9%
US Other	129	104	-19%	1049	839	-2%

Abbreviations: TFV: tenofovir; TFV-DP: tenofovir-diphosphate; ng: nanogram; mL: milliliter; fmol: femtomole; US: United States.

Supplementary Table 4: Adverse Events Leading to Study Product Discontinuation during the First Unblinded Year by Study Arm

	Cabotegravir		TDF/FTC		Total	
	N	%	N	%	N	%
Increased alanine aminotransferase	10	33.3%	12	46.2%	22	39.3%
Acute hepatitis C	5	16.7%	2	7.7%	7	12.5%
Increased aspartate aminotransferase	2	6.7%	1	3.8%	3	5.4%
Increased lipase	3	10.0%	0	0%	3	5.4%
Increased amylase	1	3.3%	1	3.8%	2	3.6%
QTc interval prolonged on electrocardiogram	2	6.7%	0	0%	2	3.6%
Acute hepatitis B	1	3.3%	0	0%	1	1.8%
Acute myocardial infarction	1	3.3%	0	0%	1	1.8%
Anal chlamydia infection	0	0%	1	3.8%	1	1.8%
Cholelithiasis	0	0%	1	3.8%	1	1.8%
COVID−19	0	0%	1	3.8%	1	1.8%
Decreased creatinine renal clearance	0	0%	1	3.8%	1	1.8%
Depression	0	0%	1	3.8%	1	1.8%
Diarrhea	0	0%	1	3.8%	1	1.8%
Drug−induced liver injury	1	3.3%	0	0%	1	1.8%
Hepatitis A	1	3.3%	0	0%	1	1.8%
Hepatitis E	0	0%	1	3.8%	1	1.8%
Hypersensitivity	1	3.3%	0	0%	1	1.8%
Nausea	1	3.3%	0	0%	1	1.8%
Non−alcoholic steatohepatitis	0	0%	1	3.8%	1	1.8%
Presyncope	1	3.3%	0	0%	1	1.8%
Gonococcal proctitis	0	0%	1	3.8%	1	1.8%
Suicide attempt	0	0%	1	3.8%	1	1.8%

Abbreviations: TFV: tenofovir; FTC: emtricitabine

Supplementary Figure 3: Median Changes in Weight (kg) from Enrollment (W0) to Week 153 (W153)

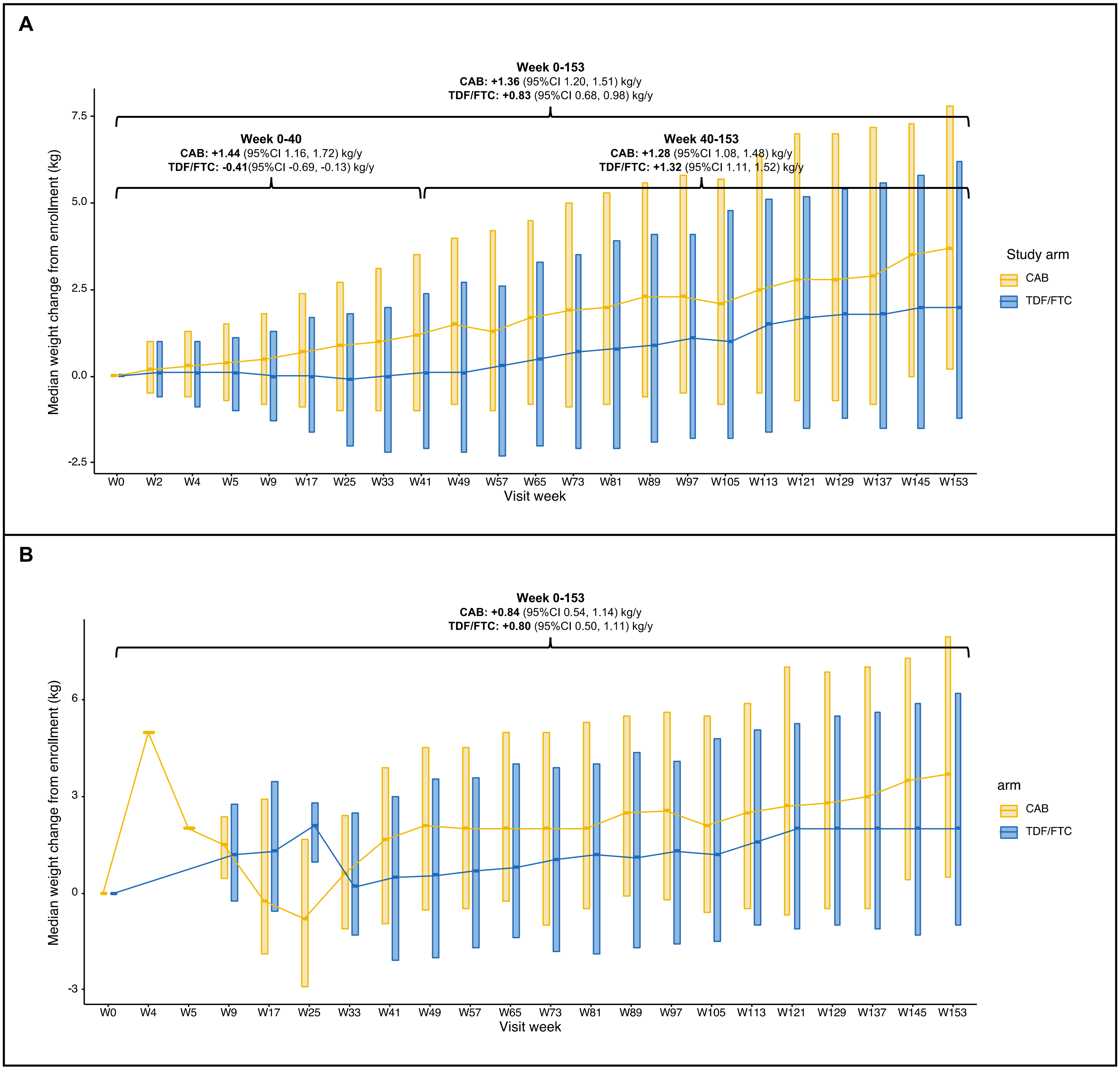

Panel A: The figure shows median change in weight from enrollment (W0) to Week 153 by study arm for the combined analysis of the blinded phase and first unblinded year. Panel B: The figure shows median change in weight from enrollment (W0) to Week 153 by study arm for first unblinded year only. Bars at indicate the median (center horizontal line) and interquartile range (IQR, top and bottom of bars). Linear mixed-effects regression analysis used to determine mean weight change.

Abbreviations: CAB, cabotegravir; TDF, tenofovir disoproxil fumarate; FTC, emtricitabine; CI: confidence intervals; kg/y: kilograms/year; IQR, interquartile range; W: week.

Supplementary Acknowledgements

UNITED STATES SITES

Alabama CRS (UM1AI069452)

Gina Deavers, Heather Logan, Catrena Johnson, Tamara James, Anna Moyana, Francine Smith, Michelle Chambers, Deon Powell, Paul Goepfert, Kamellia Safavy, E. Turner Overton

UCLA Care Center CRS (UM1AI069424)

Raphael J. Landovitz, Maricela Gonzalez, Tiara Yamamoto, Irma Franco-Gonzalez, Judith S. Currier, Jennifer J. Chang, David Goodman-Meza, Alex Iling, Michelle Simek, Aleen Khodabakhshian, Ste'von Afemata, Sonia Salas-James, Christine Jackson, Brooke Rodriguez, Melinda Kuo, Arezou Sadighi-Akha, Mariel Selbovitz, Uyen Kao, Lisa Mark, Matt Mueller, Mitch Belkin, John Cabantac, Christina Shin, Hannah Mansky, Heather Karpf, Ghassan Khoury, Maria Palmer

UCLA Vine Street Clinic CRS (UM1AI069424)

Steve Shoptaw, Jesse Clark, Michele Vertucci, William Hernandez, Susan Reed, Chris Blades, Schuyler Thomas, Demetria Villanueva, Jonathan Veloz, Sandy MacNicoll, Jennifer Baughman, Jasmin Tavarez, Page Briscol, Christina Shin, Hannah Mansky

East Bay AIDS Center (EBAC) CRS

Christopher Hall, Michele Tang, Jamie Mandelke, Tricia Smallwood, Matthew Reynolds, Ryan Anson, Jessica Horwitz, Emily Rymland, Alex Stefans, Kimery Leong, Steven Oakes, Tuan Nguyen, Gloria Pang, Myesha Kirk, Alexandra LaCorte

Bridge HIV CRS (UM1AI069496)

Al Liu, Alfonso Diaz, Allison Phillips, April Garcia, David Samayoa, Delia Molloy, Elaine Chae, Emily Schaeffer, Fiona O'Connell-Gates, Gabriella Green, Garza Asencios, Hyman Scott, Janie Vinson, John McGovern, Jonathan Van Nuys, Josephine Ayankoya, Kate McCay, Kenneth Coleman, Megan Henry, Millan Figueroa, Nicholas Ng, Nicole Walker, Patricia Von Felten, Rafael Gonzalez, Shannelle Nebre, Shadi Houshangi, Susan Buchbinder, Theresa Wagner, William Juarez, Elba Overton

Children's Hospital Colorado CRS

Dan Reirden, Betsy McFarland, Carrie Knowlton, Kim Pierce, Emily Barr, Carrie Chambers, Peaches Schweitzer, Damarcus McGill, Ibrahim Kamara, Ellen Burke, Austin Chavez, Shane Curran-Hays, Jenn Dunn, Jenny Englund, Christiane Furlong, Carrie Glenny, Paul Harding, Erin Hilgier, Alix Jones, Alisa Katai, Kay Kinzie, Myron Levin, Carol Mendrygal, Moises Munoz, Kacey Navarro, Layne Perkins, Sarah Rollo, Tori Rutherford, LaDessa Scheinost, McKenna Snyder, Michelle White-Samuels

George Washington University CRS (UM1AI069503)

Hana Akselrod, Aimee Desrosiers, Jennifer Eyrich-Ferranti, Kayley Langlands, David Parenti, Afsoon Roberts, Bitana Saintilma, Marc Siegel, Gary Simon, Nicole Swanson, Caroline Thoreson, Chelsea Ware, Daniel Anitsakis, Jeanne Jordan, Madison Lintner, Kaitlyn MacNair, Amanda Nelson, Annalise Schoonmaker, Kelly Unekis, Nicole Dornbush, Robbie Kattappurnam, Arley Hunter, Jessica Mcnube, Ryan Mouton, Cynthia Parker, Lakeisha Queen, Regina Smergalino, Nora Abdel-Gawad, Madhu Balachandran, Cheriko Boone, Aurnell Dright, Alan E. Greenberg, Nikardi Jallah, Alexander King, Carolyn Knoll, Irene Kuo, Taylor Ladson, Matt Levy, Manya Magnus, Vivitha Mani, Sophia Wozny, Hannah Yellin, Melissa Turner, Kayley Langlands

Ponce de Leon Center CRS (UM1AI069418)

Valarie Hunter, Edwin Worthington-Blount, Darian West, Carlos del Rio, Jeffrey Lennox, Rotrease Regan, Valeria Cantos, Nathan Summers, Sara Turbow, Elisa Ignatius, Colleen Kelley, John Gharbin, Karon Gaston, Catherine Abrams, Baderina Offutt, Kathy Traylor, Ossie Williams, Christin Root, Philip Powers, Rondell Jaggers, Felecia Wright, Pamela Lankford-Turner, Justin Colwell, Sree Aramgam, Tamera Franks, Tiraje Lester, Taylor Johnson, Sha Yi, Fred Ede, Damien Swearing, Derek Jobe, Chris Foster, Juliet Brown, Nursing and Lab Staff of Georgia Clinical and Translational Science, Alliance (CTSA) at Grady Memorial Hospital

Hope Clinic of the Emory Vaccine Center CRS (UM1AI069418)
Colleen Kelley, Alexis Ahonen, LaShonda Hall, Geoffrey Kamau, Harlan Smith, Rameses Frederick, Christopher Conway-Washington, Nadine Rouphael, Matthew Collins, Sri Edupuganti, Nicole Baker, Alicarmen Alvarez, Sharon Curate-Ingram, Renata Dennis, Monica Godfrey, Pamela Lankford-Turner, Shashi Nagar, Varun Phadke, Amy Sherman, Cynthia Whitney, Katie Al-Haroun, Jean Winter, Monica Godfrey, Tiraje Lester, Sha Yi, Damien Swearing, Fred Ede, Justin Caldwell, Taylor Johnson, Sreelatha Aramgam, Jianguo Xu, Machel Hunt, Ellie Butler

Adolescent and Young Adult Research at the CORE Center (AYAR at CORE)
Tope Oyedele, Sybil Hosek, Kelly Bojan, Rachel Jackson, Ixchell Ortiz-Estes, Julia Lee, Meena Malhotra, Ryan Muench, Antionette McFadden, Dorothy Rego, Hamid Bouiri, Marisol Consignado, Andre Evans, Jason Rippe, Christine Inguglia, Maria Budz

UIC Project WISH CRS
Stephanie P. Martin, Marla Schwarber, Wardrick Nealon, Rich Morrissy, Charlie Peterson, Quintin Cammack, David Moore, Rod Reyes, Richard M. Novak, Samuel Rene, Habiba Sultana

New Orleans Adolescent Trials Unit (NO/ATU) CRS
Sue Ellen Abdalian, Leslie Kozina, Alyne Baker, Brenda Andrews, Chi McKendall-Lewis, Lisa Burnett, Sean P. Sylve, Margarita Silio, Lisa Cunningham, Stephen Karl Gotzkowsky, Hamada Rady, Michelle Washington, Shanker Japa, Sandra Eloby Childress, Jasmine Fournier, Sean Williams, Trina Jeanjacques, Joseph Ricky R George Sr, Corinne Vandagriff, Laniece Thomas

Johns Hopkins University CRS (UM1AI069465)
Anne Rompalo, Ilene Wiggins, Margaret Abaandou, Shamiso Chitambira, Vivian Rexroad, Denise Wright, Nadine Brown, Desiree Nock, Charles Flexner, Kelly Dooley, Rennisse McKinley, Andrea Weiss

Fenway Health CRS (UM1AI069412)
Kenneth Mayer, Marcy Gelman, Douglas Krakower, Jessica Kraft, Taimur Khan, Julian Dormitzer, Rossi Fish, Johnathon Holmes, Sinclair Lao, Gina Cardarelli, Margarita Lewinter, Linda Ng, Ryan Earls, Kelvin Powell, Adrianna Boulin, Patrick MacDonald, Rafael Ruiz-Martinez, Shea Buckley, Janet Dargon, Julia Fleming, Brooke Travis, Ryan Tappin, Christopher Chianese, Z. Rob Moyers, Christopher Mistretta, Brooke Travis, Alison McLoughlin, Natalie Marks, May Navarra

Washington University Therapeutics CRS (UM1AI069439)
Rachel Presti, Andrej Spec, AJ Winingham, Alem Haile, Lisa Kessels, Mike Royal, John Tran, Mike Klebert, Kim Gray, Tina Robinson, Teresa Spitz, Sara Hubert, Anita Afghanzada, Laura Blair, Warren Seyfried, Trudy House, Constance Cafazza

New Jersey Medical School Clinical Research Center CRS (UM1AI069419)
Shobha Swaminathan, Christina Daliani, Jared Khan, Jamir Tuten, Travis Love, Amesika Nyaku, Michelle DallaPiazza, Rondalya DeShields, Christie Lyn Costanza, Eric Asencio, Susana Rivera, Sukhwinder Singh, Dina Meawad, Jennifer Punsal, Valerie Cadorett

Bronx Prevention Research Center CRS (UM1AI069470)
Jessica Justman, Ellen Morrison, Rita Sondengam, Ann Kahn, Rashaunna Redd, Martha Cavallo, Ana Victoria Cruz, Jawindy Swengbe, Marbella Silverio, Maria Rivas

Harlem Prevention Center CRS (UM1AI069470)
Sharon Mannheimer, Jun Avelino Loquere, Julie Franks, Orlando Rosario, John Barazzuol, Cassia Wells, Yael Hirsch-Moverman, Joshua Hinkson, Robert Gamboa, Ibrahima Cisse, Darien Thomas, Joshua Bautista, Insiya Taj, Aprielle Wills, Jaron Cook

New York Blood Center CRS (UM1AI069470)

Hong Van Tieu, Sauda Muhammad, Inja Noh, Jenny Yee-Levin, Maria Veronica Sanchez, Ruth Santos, Kiwan Stewart, Angela Lomax, Tarashon Broomes, Geneva Ortiz, Mahtab Sheikh, Margarita Ashkinazi, Jorge Soler, Jay Loeffel, Jonathan Washington, DaShawn Usher, Eddie Bravo, Gabrielle Figueroa, Vijay Nandi, Stephanie Kung, Breanne Baez, Joie Cooper, Bart Buurman, Elsie Marius, Chantel Fletcher, Rashaad Banks, Ilyas Tezerbaev, Arialdy Chavez, Christina Barron, Kipa Sherpa, Joseph Kim, Jeffrey Li, Victoria Frye, Debbie Lucy, Tasha Vazquez, Nikki Englert, Cynthia Yee, Major Nesby, Beryl Koblin

<u>Weill Cornell Chelsea CRS (UM1AI069419)</u>
Trip Gulick, Grant Ellsworth, Tim Wilkin, Valery Hughes, Shaun Barcavage, Todd Stroberg, Briain Mangano, Wayne Burns, Sophia Alvarez, Genesis Rodriguez, Catherine Jerry, Tiina Ilmet

<u>Chapel Hill CRS (UM1AI069423)</u>
Christopher Hurt, Cindy Gay, David Wohl, Joe Eron, Cheryl Marcus, Susan Pedersen, Noshima Darden-Tabb, Durham Health Department, Lincoln Community Health, Felton T., Correll M., DeAndra B, Gordon H, Brian G., Kenneth F., JT, DePris P., Tania Hossain, Nazneen Howerton, Felicia Barriga Munante, Catherine Kronk, Tracy Eldred, Abigail Riddick, Kalina Grimm, Javacia Jackson, Becky Straub, Miriam Chicurel-Bayard, Matthew Newell, Donna Pittard, Maggie Harman, Maria Stetson, Betelehem Shenbulo, Gabrielle Evans, Alexandra Roos, Brittney Soderman, Linda Manor, Maria Bullis, Amy James Loftis, Nicole Maponga, Bill Wolf, Paul Alabanza, Takesha McMillion, Laura Finerty, Dana Lapple, Janette Goins, Marie Oriol, Grace Tillotson

<u>Greensboro CRS (UM1AI069423)</u>
Cornelius Van Dam, Robert Comer, Kim Epperson, Lisa Dasnoit, Laura Keck, Marlene Allen, Caitlyn Delgado, Danielle Guilbeault, Alison Grimsley, Michelle Porter, Correll McRae, Bridget Thomas, Luwam Debru, Elisha Epperson, Tamika Yarborough, Maria Stetson, Charles Hansen, Dan Ferguson

<u>Cincinnati CRS (UM1AI069501)</u>
Carl Fichtenbaum, Moises Huaman Joo, Jaasiel Chapman, Jenifer Baer, Eva Whitehead, Michelle Saemann, Sarah Trentman, Linda Hinds, D'Vaughn House, Michele O'Neill, Brenda Miller, Elizabeth Costea, Eric Mueller, Anissa Moussa, Geronimo Feria Garzon, Sharon Kohrs, Gabrielle Cook, Sierra Bennett, Marlene Petrie, Lyndsey Armor

<u>Ohio State University CRS (UM1AI069494)</u>
Jose A. Bazan, Susan L. Koletar, Brian Greenfelder, Kathy Watson, Rabia Khan, Deliann Campbell, Jan Clark, Heather Harber, Isaac Navar, David Andrist, Lindsay Summers, Aya Vernon, Andrea Vietti

<u>Penn Prevention CRS (UM1AI069534)</u>
Ian Frank, Deb Dunbar, Jie Ho, Nicole Kordziel, Tameka Matthews, Chris Chianese, Gillain Constantino, Petra Alexander, Dana Brown, Annet Vogel, Dan Mangini, Aeryanah Bryant, Richard Tustin III, Nicole Sundo, Laura Schankel, Kate Kearns, Kenneth Rockwell, Michele Wisniewski, Susan Carney, Jason DeWitt, Melanie Furjanic, Nancy Tang, Thomas Han

<u>St. Jude Children's Research Hospital CRS (UM1AI069536)</u>
Aditya Gaur, Patricia Flynn, Katherine Knapp, Nehali Patel, Shelley Ost, Sally DiScenza, Carla London, Ramona Burgess, Tara Brown, Hannah Allen, Mary Dillard, Kim Deluca, Christina Owens, Angela London, Jenny Knych, Susan Carr, Camille Smith, Robbin Christensen, Charles Longserre, Julie Richardson, Kirk Knapp, Andrea Stubbs, DeMarcus Jones, Ayeisha Cole, Susannah Keck, Hattie Stewart, Vijay Paladugo

<u>Houston AIDS Research Team (HART) CRS (UM1AI069503)</u>
Roberto Arduino, Jonatan Gioia, Sofia Lupo, Maria Laura Martinez, Olga Ortiz, Martine Diez, Abdul Gabisi, Celia Fenceroy, Maria Florencia Martins

ARGENTINA SITES
<u>Fundación Huésped CRS</u>

Pedro Cahn, Omar Sued, Valeria Fink, Carina Cesar, Patricia Patterson, Inés Figueroa, Luciana Spadaccini, Claudia Frola, María V. Iannantuono, Carolina Pérez, Marcelo Gismondi, Herman Ludvik, Daniela Parera, Sergio Sciannameo, Mirna Gaona, Ana Gun, María R. Nemenman, Natalia Panis, Jonathan García, Lara Fernández, Horacio Beylis, Rocío Lada, Luisina Fernández, Agustina Nuñez, Yamila Martinez, Lara Vladimirsky, Emanuel Fojo, Verónica Viggiano, Fabiana Enjamio, Agustina Arguello, Emanuel Dell Isola, Mariana Pugliese, Inés Aristegui, Mariana Duarte, Solange Fabián, Nadir Cardozo

Hospital General de Agudos JM Ramos Mejía
Marcelo Losso, Laura Moreno Macias, Florencia Crupi, Guillermo Viloria, Patricia Burgoa, Angel Parlante, Samanta Arrigorriaga, Nadia Longo, Cecilia Abela, Valentina Losso, Gonzalo Dieguez Gaviola, Sofia Frenkel, Leonardo Perelis, Norberto Selasco, Stella M. Timpano

BRAZIL SITES
Hospital Nossa Senhora da Conceição CRS (UM1AI069424)
Andréa Cauduro de Castro, André Luiz Machado da Silva, Breno Riegel Santos, Chaiane Zucchetti, Consuelo Freitas Perez, Dimas Alexandre Kliemann, Eliete Sampaio Fleck, Elizabeth Silva de Magalhães, Fábria Chiarani Campos, Gabriela Duarte Ramos, Fernanda Kuhn, Henry Carmona Kammler, Kelin Roberta Zabtoski Piovesana, Lisdiê Constante Machado, Magnus Cassio Trindade de Melo, Mara Liane Rieck Silveira, Marcelo Edison Vieira de Almeida, Maria Cristina Chaves Seter, Maria Lourdes Somagal Turella, Mariana Riegel de Pádua Simon Moesch, Marineide Gonçalves de Melo, Mickaela Fischer Silva, Priscila de Lima Pelaez, Rita de Cássia Alves Lira, Roberta Fleck dos Santos, Rui Flôres, Salete Maria Zabtoski, Teresinha Joana Dossin, Vera Lucia de Souza Soares, Julio Barros, Ana Vidal, Raquel Trassante, Sandra Gomes, Thiago Khan, Eduardo Lima, Cleo Silva, Thiago Charme, Julio Barros

Instituto de Pesquisa Clinica Evandro Chagas (IPEC) CRS (UM1AI069476)
Beatriz Grinzstejn, Valdilea Veloso, Lara Coelho, Egydio Sampaio, Debora Barreto, Juliana Maia, Leonardo Paiva, Desiree dos Santos, Rodrigo Escada, Sandra Wagner, Lucimar Salgado, Tamiris Baiao, Jéssica Felix, Sandro Nazer, Vladimir Mota, Gisele Hottz, Cleber Magalhães, Kelly Gama, Claudio Junior, Ericka Mascarenhas, Thamara de Sousa, Thiago Torres, Valeria Ribeiro, Luana Marins, Giovanna Costa, Robson Pierre, Cristina Jalil, Eduardo Netto, Daniel Waite, Daniel Bezerra, Josias Freitas, Laylla Monteiro, Marcos Araujo, Tania Krstic, Tania Brum, Flavia Lessa, Luiz Camacho, Ana Paula Gomes, Natalia Maia

Centro de Pesquisas Clínicas IC-HCFMUSP CRS
Esper G. Kallás, Natália Cerqueira, Ricardo Vasconcelos, Zelinda Nakagawa, Leandro Concolato, Maria Candida Dantas, Helena Tomiyama, Karine Silva, Rosangela Silva, Denivalda Araújo, Daniel Bertevello, Claudia Tomiyama, Aline Anjos, Maria Angélica Neves, Issler Morais, Daniel Barros, Michelle Barboza, Denise Sales, Gislayne Lima, Edson Guedes, Thiago Sabino, Luiz Zanella, Renata Oliveira, Raphaela Fini, Piero Mori, Camila Donini, Fabio Ghilardi, Mary Helen Morais, Gustavo Rezende, Luana Santos, Anna Silva, Carlos Moreira, Simone Tenore, Elizabeth Pereira, Neivaldo Fiorin, Gabriel Borba, Camila Rodrigues, João Magri, Nailson Virgens, Geovanna Farias, Angelas Freitas, Edmilson Medeiros, Francisco Felix, Bárbara Henriques, Alberto Tomiyama, Marilia Antonio, Carolina Lobo, João Araújo, Maria Pereira, Taynan Rocha, Mariana Sauer, Jaqueline Carvalho, Marcia São Pedro, Athos Souza, Carlota Miranda Paredes, Pedro Henrique Figueiredo, Bruna Moreira, Verônica Silva, Natacha Cerchiari, Rosimeire Zaboto, Júlia Tronco, Ferdinando Menezes, Cristiane Amorin, Marjorie Rapozo, Priscila Souza, Anuradha Barreto

Centro de Referência e Treinamento DST/AIDS CRS
José Valdez Ramalho Madruga, Roberta Schiavon Nogueira, Aline Carralas Queiroz de Leão, Álvaro Furtado Costa, Ana Caroline Coutinho Iglessias-, Camila Albuquerque Moraes, Clara Vidaurre Mendes, Érika Maria do Nascimento Kalmar, Fábio Luis Nascimento Nogui, Karina Takesaki Miyaji, Lucas Rocker Ramos, Maria Silvia Biagioni Santos, Maisa Miguel Benette, Patricia Rady Muller, Suzana Toledo da Silva Leme, Aline Barnabé Cano, Maura Regina da Silveira, Priscilla de Lima e Menezes, Ricardo Augusto Braga Castro, Goher Lima Gonzales, Sâmia Silveira Souza Teixeira, Adriana

Balduino de Azevedo, Luciana Satriano Baptista de Moura, Fábio Rodrigues da Silva, Herla Ignez G. de Souza, Débora Lopez Teixeira Lopes, Kelly Vieira, Roberta Bocalon, Sandra de Araujo, Rosaria Maria Martinez, Maria das Graças Costa Cavalcante-, Wylliam Magnum Telles Dias, Lorena Henn, Vinicius Francisco da Silva, Valvina Madeira Adão, Dirce Candida de Assis, Elvira Maria Ventura Felipe, Elaine Gaeter Gonzalez Pinto, Mariza Fumiko Nakae, Adriana Claro de Oliveira, Vilciani Borges de Souza, Maria Cristina Silva, Carué Contreiras, Cristiane Sales Amorim, Ana Paula Loch, Luciene da Silva Arrifano, Ana Tercia Nascimento, Gabriela Guizi Pereira, Cintia Dellaqua, Natália Amdi Cesari, Lais Braga Soares, Eduardo Prevelato Athayde Junior, Daniel Gleison Carvalho, Cleuza Santos Lopes, Rosa Blanco Matos, Márcio Bertozzi, Beatriz Zveibil

PERU SITES

Asociación Civil Selva Amazónica (ACSA) CRS (UM1AI069438)

Martin Casapia, Juan Carlos Hinojosa, Ana Rimachi Quiroz, Lucía Ruiz, Carlos Vela, Tania Flores Metzger, Glendy Chamoli, Dori Pashanaste, Jessica López, Aldo Isla, Brenda Urday, Claudia Ramos, Alejandro Quiroz, Karen Campos, Javier Achong, Nieves del Aguila, Mexi Puerta, Silvia Marin, Mónica Chauca, Ceily Silva, Lilia Pinedo, Rocío Jaramillo, Luisa Mowerg, Patsy Torres, Cristina Vela, Carolina Arredondo, Dennis Vela, Andre Cubas, Gonzalo Grandez, Renato Guedes, Jimmy Panduro, Claudia Rojas, Raul Gonzales, Marco Calixtro, Claudia Vargas, Rocio Angulo, Katherine Lozano, Dacil Angulo, Veronica Araujo, Francisco Ruiz, Luis Bolivar, Roxana Pezo, Libby Diaz, Lucero Rodriguez

Barranco CRS (UM1AI069438)

Jorge Sanchez, Javier Lama, Javier Valencia, Rosa Infante, Mey Leon, Ernesto Alayo, Joshua Paz, Akemi Matsuno, Jorge Gallardo, Milagros Matta, Saul Levy, Francesca Cordano, Melany Esteban, Manuel Villaran, Yesika Magallanes, Roxana Vargas, Maria Mamani, Fany Rosas, Rosa Blas, Jessica Alva, Aura Jara, Jose Eleazar, Clery Palacios, Helen Chapa, Dalila Salazar, Edith Muñoz, Areli Deliot, Diego Rojas, Adela Huaman, Martin Llancare, Mirna Garrido, Carlos Caceres, Heydee Diaz, Kevin Cruz, Dayhan Contreras, Miguel Angel Chirre, Kevin Puellas, Felipe Vilcachagua, Pio Huaycho, Egdin Amias, Arturo Sueldo, Gian Carlo Salazar, Lucho Castro, Rosario Leon, Carmela Ganoza, Cecilia Chang, Lily Ganaha, Ricardo Alfaro, Brenda Mauricio, Carmen Salinas, Maria Del Carmen Suarez, Giovanna Barrios, Nieves Castillo, Consuelo Regalado, Virginia Riojas, Elisabeth Astupina, Yerica Valenzuela, Jose Mejia, Remo Gonza, Sandy Perez, Yorka Alaria, Alejandra Flores, Raul Inocente, Gladys Cabracancha, Hector Garriazo, Martin Patiño, Aron Trujillo, Karen Villanueva, Tula Quispe, Judith Jajaycucho, Soledad Vargas, Cristina Angeldonis, Gladys Chacon, Rocio Yupanqui, Jakeline Alcazar, Luis Limo, Diana Morales, Valeria Fulqui, Eduardo Ruiz, Peter Brandes

Centro de Investigaciones Tecnologicas, Biomedicas y Medioambientales (CITBM) CRS

Jorge Gallardo-Cartagena, Jorge Sanchez, Fanny Rosas, Carlos Medrano, Karin Sosa, Karla Tafur, Martin Moreno, Pamela Ramirez, Juan Montenegro, Claudia Tirado, Armando Piedra, Julio Yonamine, Akemi Matsuno, Gustavo Grandez, Joshua Paz, Patricia Kaway, María Sanchez, Dora Guarniz, Leslie Arancibia, Anibal Vilca, Aura Jara, Narda Montoya, Raquel Espinoza, David Amiel, Sheimy Abad, Yulios Rodriguez, Viviana Ghiglino, Deisy Sanchez, Carlos Romero, Jonathan Zamora, Diego Torres, Hugo Sanchez, Julio Ortiz, Daniel Alva, David Velasquez, Ivan Gonzales, Miguel Gonzales, Fabricio Sanchez, Yolanda Vidal, Irma Izquierdo, Julissa Juarez, Mishiko Sato, Miguel Morales, Roselena Godos, Jose Calienes, Angelo Motta, Carmen Astuvilca, Claudia Arbañil, Cristina Angeldonis, Diana Morales, Gustavo Calleja, María Carrión, Ricardo Alfaro, Richard Negron, Jose M. Guevara, Andrea Paredes, Magnolia Luque, Lenin Silva, Andrea Bermudez, Rosa Zanabria, Ruller Soto, Chilbernan Callirgos, Lucas Sevilla, Guillermo Cajahuanca, Silvana Garcia, Sandy Perez, Sheyber Lifonzo, Yelitza Carhuaz, Diego Moreno, Paula Tamariz, Jose Mesia, Alys Chavez, Luis Rodriguez, Nancy Salcedo

San Miguel CRS (UM1AI069438)

Lissette Rodríguez Pablo, Cecilia Correa Celi, Milagros Sabaduche Rosillo, Roder Flores Sánchez, Richard Francis Teran Guevara, Manuel Neyra Montaño, John Mac Rae Thays, Martin Lacherre Vargas, Carmen Sanchez Torres, Liliana Vallejos Quinto, Ricardo Fernando Quiroz Céspedes, Diana Durand Pinedo, Karina Tacza Mariño, Diana Rojas Huasasquiche, Bertha Talaverano Gonzales, Pedro Gonzales Saenz, Lady Albornos Aliaga, Hector Javier Salvatierra Flores, Karina Pareja Luna, Diego Quiroz Farfán, Kevin Hiro Callata Encarnación, Esperanza Yuriko Vicente Lozano, Daniela Zegarra

Ojeda, Gladys Cabracancha, Gian Carlo Yamir Salazar Chavez, Egdin Amias Macuyama, Yuliana Carlos Girao, Luis Oscar Castro Asencio, Maritza Margot Baca Lazo, Bertha Mercedes Talaverano Gonzales, Maria Gabriela Bullon Dominguez, Kattia Eloisa Montes Rodriguez, Christian Teófanes Rojas Contreras, Juan Steve Riojas Arnao, Martin Isaias Lacherre Vargas, Vanessa Galvez Zeballos, Martin Gutierrez Flores, Manuel Neyra Montaño, Diego Ismael Quiroz Farfan, Carla Josefina Porcile Chavez, Carmela Lucila Gonoza Arenas, Maria Cecilia Chang Ching, Brenda Mauricio Nisshioka, Daniela Del Carmen Zegarra Ojeda, Katherine Milagros Charri Macassi

Via Libre CRS
Aileen Castellares Sotelo, Alberto Florez Prada, Alex Barrantes Requiz, Ana Arroyo Nazario, Anderson Troya Paredes, Angela Madueño Delgado, Carlos Anton Talledo, Cesar Cardenas Aguirre, Cesar Flores Maldonado, Consuelo Tristan Ramirez, Daniel Dextre Zurita, Diego torres Nolasco, Edgar Grados Chagray, Eduardo Cachay Cuizano, Edwin Palomino Alvarez, Enrique Avila Liendo, Enrique Oscategui Ricapa, Felix Echeverria Cuba, Fernando Roman Chingne, Fernando Soto Frebres, Fernando Vilchez Torres, Frankin Amasifuen Fernandez, Geannina Sanchez Valladares, Giulianna Estrada Franco, Grecia Geri Romero, Gustavo Grandez Castillo, Gustavo Woll Buse, Hector Menacho Montenegro, Irving Diaz de La Cruz, Jean Patiño Ramos, Jennifer Tolentino Ramirez, Jessica Gutierrez Lazaro, Jose Cardozo Rodrigues, Jose Castro Chuquillanqui, Jose Ipanaque Sandoval, Jose Mendoza Paredes, Jose Vasquez Cerro, Julio Ortiz Guevara, Junior Gonzales Cueto, Karina Espinoza Maza, Karla Suarez Bendezu, Lizzet Martinez Dionisio, Luis Mesias Bracamonte, Luisa Siguas Carhuaricra, Maria del Rosario Fernandez Villanueva, Mario Sinti Ycochea, Martin Ramirez Quijandria, Mirian Huanachea Polanco, Narendar Kumar, Oscar horna Garcia, Pedro Hipolito Quispe, Raul Lama Pascarella, Renato Bobadilla Leon, Robinson Cabello Chavez, Rocio Perez Gonzalez, Rosemarie Ayarza Escobar, Rudy Huaman Edery, Ruth Delgado Escalante, Sonia Montesinos Calleja, Susan del Pilar Chavez Gomez, Tania Blacido Pascual, Victor Hugo Moquillaza, William Tipiani Ramos, Yaniz Carol Lazo Callupe, Yerica Valenzuela Garcia, Yvett Pinedo Ramirez

THAILAND SITES
Silom Community Clinic CRS
Eileen F. Dunne, Taweesap Siraprapasiri, Chaiwat Ungsedhapand , Wasin Matsee, Anupong Chitwarakorn, Andrew Hickey, Christie Vu, Benjamaporn Chaipung, Anuwat Sriporn, Chidanan Krasan, Jiriporn Nantavishit, Poomin Nongchang, Jirawat Suksamosorn, Warunee Thienkrua, Chariya Utenpitak, Dararat Worrajittanon, Siriphak Pongthai, Wannee Chonwattana, Santi Winaitham, Wichuda Sukwicha, Somsak Yafant, Rinda Wongbenchaporn, Patnaree Oungprasertgul, Phanurassamee Sittidech

Thai Red Cross AIDS Research Centre (TRC-ARC) CRS (UM1AI069418)
Nittaya Phanuphak, Praphan Phanuphak, Kiat Ruxrungtham, Nipat Teeratakulpisarn, Pongsakorn Surapuchong, Supanat Thitipatarakorn, Tanat Chinbunchorn, Ashmanie Reshmie Ramautarsing, Siriporn Nonenoy, Napasawan Chinlaertworasiri, Patsadaporn Narawin, Thanyapat Chaya-ananchot, Piranun Hongchookiat, Thunyasuta Prasit, Supawan Promkaewto, Achiraya Chanta, Khanittha Janthawilai, Aphakan Klinsukontakul, Shareefah Hayikateh, Warisa Tathian, Kritima Samitpol, Kittichai Promjantuek, Atipan Phoungphu, Sumitr Tongmuang, Kantanat Kanetrat, Sasiwimol Ubolyam, Apicha Mahanontharit, Theera Dalodom, Anuntaya Uanithirat, Chavalun Ruengpanyathip, Kittima Sakorn, Peeraporn Kaewon, Suwittra Chaemchuen

Chiang Mai University (CMU) HIV Prevention CRS (UM1AI069399)
Suwat Chariyalertsak, Natthapol Kosashunhanan, Taweewat Supindham, Nuntisa Chotirosniramit, Patcharaphan Sugandhaveha, Amaraporn Rerkasem, Quanhathai Kaewpoowat, Pongpun Saokhieo, Ratchanok Songsupa, Amornrat Yangnoi, Rattanaporn Intarawiwarat, Pimsuda Panyawong, Tharida Chaladtunyakorn, Kachaporn Yosbud, Wareerat Kaewphokha, Thanyarat Wannasopha, Suprisara Noomthongkum, Sobhon Bipodhi, Pachara Dechasakulpan, Warunee Jit-aree, Chiraphorn Kaewkosaba, Piyathida Sroysuwan, Boonlure Pruenglampoo, Umpava Timpan, Nittaya Chuenchop, Sineenart Taejaroenkul

VIETNAM SITE

Yen Hoa Health Clinic CRS

Vivian Go, Tran Viet Ha, Tran Van Sau, Tran Quoc Hung, Le Minh Giang, Hoang Duc Hanh, Do Thi Thu Ha, Ngo Thi Kim Hoa, Le Thi Thanh, Nguyen Quang Vu, Do Nguyen Thu Trang, Tran Thi Thu Hoai, Luong Thi My Ly, Le Thi Thuc Anh, Nguyen Phuong Loan, Lam Thi Binh, Le Thi Ngoc Anh, Do Thi Ngan, Tran Lan Chi, Nguyen Ngoc Son, Do Thu Thao, Kieu Duy Anh, Le Trung Kien, Le Minh Khoi, Tran Van Tai, Dang Thi Ly Nham, Nguyen Thuy Linh, Nguyen Hai Linh, Nguyen Thi Thu Huong, Pham Thi Huong, Nguyen Thanh Hai, Tran Thi Hoa, Tran Van Huong, Dau Sy Nguyen, Ha Nhu Bach, Do Viet Hiep, Nguyen Bao Ngoc, Vu Ngoc Thang, Nhu Le Kien, Bui Ngoc Dong, Nguyen Xuan Khanh, Tran Tat Dat, Truong Thai Phuong, Vu Van Truong, Bui Tuan Anh, Teerada Sripaipan, Susan Pedersen, Amy James Loftis, Tania Hossain, Becky Straub, Catherine Kronk, Pham Tien Hoa, Chu Viet Anh, An Thanh Ly, Nguyen Ngoc Chiu, Pham Ngoc Hao, Nguyen Minh Dung, Trinh Ngoc Duy, Nguyen Thi Bich Hanh, Nguyen Duy Binh, Do Kim Cuong, Nguyen Quang Tho, Luc Quach Binh Guong, Khuat Duy Nam

SOUTH AFRICA SITE

Groote Schuur HIV CRS (UM1AI069519)

Keren Middelkoop, Doerieyah Reynolds, Brandon Perumaul, Daniel Ndzuzo, Nehemiah Advance Simango, Monica Vogt, Melanie Maclachlan, Zareena Dhansay, Melina Carr, Phyllisty Smith, Nomvuselelo, Kalolo, Bianka van der Westhuizen, Yashna Singh, Sibulele Nozewu, Lameze Abrahams, Siyabonga Nombali, Amelia Mfiki, Nobafundi Dondolo, Philosophy Bangira, Siyabonga Mdlalo, Xolani Mvula, Stanley October, Precious Garnett, Peter Chodacki, Nontle Madinga, Catherine Orrell, Vanessa Simkins, Fahkri Williams, Mary Chidanyika, Richard Kaplan, Francois Cilliers, Rochel Jacobs, Nomawethu Ngcukana, Luyanda Sebe, Asanda Tolashe, Sheetal Kassim, Anna Cross

ADDITIONAL PARTNERS

Gilead Sciences

Rich Clark, Brenda Ng, Richard Haubrich, David Piontkowsky, Cal Cohen, Joel Gallant, Christian Callebaut, Moupali Das, Jared Baeten, Chris Nguyen, Fernando Bognar

ViiV Healthcare

Amy Cutrell, Britt Stancil, Susan Ford, Shanker Thiagarajah, Navdeep Thoofer, Beth Austin, John Pottage, Subrina Kaur Baghri, Carolina Acupuil
